# Signal Enhancement in Immunoassays via Coupling to Catalytic Nanoparticles

**DOI:** 10.1021/acssensors.5c00995

**Published:** 2025-05-20

**Authors:** Christy J. Sadler, Jan P. Sandler, André Shamsabadi, Leah C. Frenette, Adam Creamer, Molly M. Stevens

**Affiliations:** † Department of Materials, Department of Bioengineering, Institute of Biomedical Engineering Imperial College London, London SW7 2AZ, U.K.; ‡ Department of Physiology, Anatomy and Genetics, Department of Engineering Science, Kavli Institute for Nanoscience Discovery, 6396 University of Oxford, Oxford OX1 3QU, U.K.

**Keywords:** gold nanoparticles, catalytic nanoparticles, immunoassays, lateral flow immunoassays, signal amplification

## Abstract

Early diagnosis is vital for effective disease management, selection of appropriate treatment regimes, and surveillance and control of disease transmission. There is a growing need for point-of-need diagnostic platforms, such as lateral flow immunoassays (LFIAs), to reduce healthcare burdens, particularly in low-resource settings. However, LFIAs often suffer from inadequate sensitivity and exhibit limited dynamic ranges, leading to late-stage diagnosis or misdiagnosis. Here, we present a signal enhancement platform for use in both plate- and paper-based immunoassays, based on the formation of a coupled nanoparticle network. We demonstrate the coupling of an antigen-targeting detection probe with a secondary, catalytically active nanoparticle by utilizing secondary antibody interactions. Here, we show that signal enhancement is achieved through two functional mechanisms: network formation, facilitated by the secondary nanoparticle increasing the relative concentration of nanoparticles immobilized at the test zone; and the inclusion of catalytically active nanoparticles, which catalyze the oxidation of a chromogenic substrate at the test zone. Through this approach, we yielded a 40-fold improvement in the limit of detection (LOD) using 40 nm gold nanoparticle detection probes in spiked pooled human saliva. Further, the signal enhancement platform can be utilized alongside a range of detection probes, including gold nanoparticles, commonly employed for use in LFIAs. This work concludes by showcasing that the signal enhancement mechanism is compatible for use with complex sample matrices, such as human saliva.

## Graphical abstract

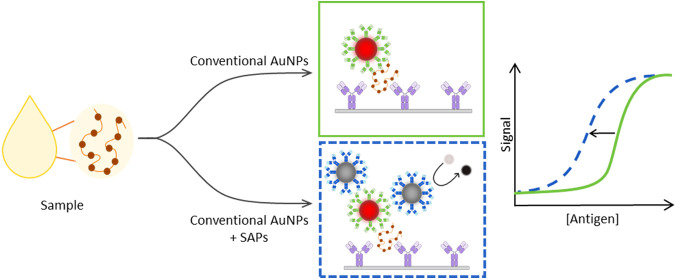

The decentralization of healthcare has, in part, gained traction due to the availability and acceptance of point-of-care biosensors.^1,2^ Such biosensors must feature straightforward sample collection protocols, a user-friendly operation, and the ability to produce rapid results that can be read without the need for specialized equipment, as outlined by the REASSURED criteria.^3,4^ The most commonly utilized point-of-care biosensors are lateral flow immunoassays (LFIAs).^5^ Currently available LFIAs include pregnancy tests (hCG detection in urine, with a naked-eye or digital readout),^6^ Covid-19 rapid diagnostic tests (viral protein detection in saliva or nasopharyngeal swab, with a naked-eye readout),^2^ and cholera tests (detection of lipopolysaccharides on bacteria surface from stool samples with a naked-eye readout).^7^

LFIAs rely on specific interactions between affinity agents (typically antibodies), nanoparticles and target biomarkers to produce an interpretable signal. Briefly, capture immunoreagents are typically immobilized onto a nitrocellulose membrane to form test and control lines. Nanoparticles (such as colored latex beads or gold nanoparticles (AuNPs)) are functionalized with immunoreagents to form detection probes. These probes are exposed to the patient sample, where they interact with and bind to the target biomarker. Complexes consisting of the detection probe and target biomarker are then immobilized on the test line producing a signal that is typically read by the naked eye.^2,5,8^ Components of the LFIA can be modulated to tune the analytical performance of the developed assay, ensuring that the clinically relevant range of target biomarker can be detected (typically from nM to fM for protein biomarkers). Specifically, this can include the choice of affinity agent pairs, nanoparticle probes and functionalization conditions, and the composition of membranes.^5^ Further, the format of LFIAs can be varied according to the specific use case and need. This includes the use of half dipstick LFIAs, whereby the nanoparticles are added to the test strip along with the sample, which is commonly used when developing new assay technologies. Other test formats, such as dipstick and full cassette based LFIAs, involve the drying of nanoparticle probes onto conjugate pads. These have advantages for use by patients directly due to the reduced need for end user engagement to operate the test.

Due to the nature of commonly used detection probes, LFIA readouts are typically qualitative, indicating only the presence or absence of a particular biomarker through generation of a visible test line.^5,9^ However, the use of LFIAs for quantitative biomarker detection is becoming increasingly desirable and is necessary to expand the impact of point-of-care testing.^10^ In order to achieve quantification, the developed LFIA must have a sufficiently broad dynamic range, defined as the concentration span between the lowest biomarker concentration that can be detected and quantified (with a given confidence level) and the highest concentration at which an assay’s response is linear. For LFIAs with naked-eye readout mechanisms, the dynamic range is limited by visual detection limits at low target biomarker concentrations, and signal saturation and the hook effect at high concentration levels.^11^ This can present challenges for the detection of biomarkers that have a large range of clinically relevant concentrations. One example is the semiquantitative detection of C-reactive protein (CRP) using naked-eye readouts, in which multiple test lines are employed to estimate CRP concentration.^12,13^ The clinically relevant range of concentration for CRP spans a broad dynamic range (1 to ≥ 250 mg L^–1^). This presents a challenge for quantification using traditional sandwich LFIAs due to the presence of the hook effect at high CRP levels.^11^ To overcome this, multiple test lines are commonly introduced into the LFIA test strip.^11,13^ Commercially available LFIAs can detect CRP levels at <10 mg L^–1^; 10–40 mg L^–1^; 40–80 mg L^–1^; >80 mg L^–1^ (bioNexia CRPplus) through the employment of three spatially distinct test lines.^12^ However, this method can increase the manufacturing costs associated with the production of the LFIA test strip due to the increased amount of affinity agent required for the assay.^11^ Further, dilution of the patient sample can be used to ensure the concentration of target biomarker in the sample falls within the dynamic range of the developed LFIA. However, the dilution of a patient samples reduces the net target biomarker concentration, increasing the assay detection limit. Care should therefore be taken when utilizing sample dilution as a method to broaden the dynamic range of an assay.

A further limitation of LFIAs is their varying analytical sensitivity and specificity, particularly in comparison to gold-standard molecular diagnostic techniques, such as PCR.^14^ While current sensitivities may be sufficient for highly abundant or upregulated biomarkers (e.g., viral protein levels in the latter stages of infection), they pose a bottleneck for detecting biomarkers at the earliest stages of disease.^2,15^

Efforts have been made to develop signal amplification strategies that can be readily integrated into existing LFIA architecture.^16^ In order to generate a visible signal, detection probes must accumulate on the test line. Work by Khlebtsov et al. aimed to quantify the number of AuNP detection probes that must be immobilized on the test line to produce a visible signal. It was found that for 40 nm AuNPs, a minimum of 3.78 × 10^6^ particles mm^–2^ was required to distinguish the test line from the background and generate a minimum visible signal. This sets a theoretical LOD of 18.9 amol (assuming 1:1 binding ratio between the AuNP conjugate and antigen).^17^ To enhance the signal generated at the test line, research has focused on increasing the number of nanoparticles immobilized on the test line through the formation of nanoparticle networks.^18–21^ These strategies utilize AuNP detection probes, coupled to signal amplifying AuNPs via a number of mechanisms, such as the use of secondary antibodies,^21^ biotin–streptavidin interactions,^20^ binding to generic blocking proteins,^19^ and the hybridization of single stranded DNA.^18,22^ These approaches have demonstrated signal enhancement ranging from 3 to 100-fold. Further work has explored modifications to the nanoparticle probe and the readout mechanism. In particular, catalytically active nanoparticles, such as enzyme-mimicking platinum nanocatalysts (termed PtNCs), have shown promise in improving sensitivity and extending the dynamic range of assays with naked eye readouts.^23,24^ These nanoparticles produce an enhanced colorimetric readout due to their high contrast with the nitrocellulose membrane. The generated test line intensity is amplified further by utilizing the oxidation of chromogenic substrates, such as 3,3′,5,5′-tetramethylbenzidine (TMB), to produce an insoluble precipitate. This precipitate accumulates at the test (and control) line, amplifying the signal.^23^

Beyond naked-eye readouts, the measurement of fluorescence intensity has shown promise for the quantitative detection of low abundancy target biomarkers. This includes the use of fluorescent nanoparticles, such as quantum dots and fluorescent liposomes, functionalized with affinity agents.^25–27^ To further improve the sensitivity of fluorescence-based readouts, composite nanoparticles have been developed, such as magnetically retrievable fluorescent nanoparticles that facilitate sample concentration and enrichment, prior to running the LFIA.^26,28^ Moreover, fluorescent nanodiamonds have been shown as promising quantum materials for use in fluorescent LFIAs,^29,30^ offering improved sensitivity (58-fold) when used in place of AuNPs.^29^ However, these approaches come with limitations, owing to the need for specialized readers to interpret test results.

In this work, we present a signal enhancement strategy based on the coupling of antigen-targeting detection probes to catalytically active signal amplifying nanoparticles via secondary antibody interactions. Here, we functionalize nanoparticles with antigen-targeting primary antibodies against SARS-CoV-2 nucleocapsid (N) protein as a model antigen to produce detection probes. Signal amplifying nanoparticles (SAPs) are functionalized with the corresponding secondary antibodies. The SAPs consist of functionalized platinum nanocatalysts, leading to signal enhancement through aggregate network formation and inclusion of catalytically active nanoparticle species. We demonstrate that this signal enhancement platform can be used with a range of detection probes (40 and 80 nm AuNPs, and PtNCs), in both plate- and paper-based assays. Finally, we showcase the platform’s ability to produce signal enhancement when used with complex samples, such as using pooled human saliva (from ≥ 3 donors) spiked with recombinant SARS-CoV-2 N protein antigen.

## Results and Discussion

### Synthesis of Nanoparticle Conjugates

In order to produce nanoparticles with antigen targeting ability, detection nanoparticles were functionalized with primary mouse monoclonal antibodies against SARS-CoV-2 N protein (N9547, Meridian Bioscience, supplied by Biorbyt). This was achieved via passive adsorption to generate functional detection probes. Three nanoparticle species were utilized as detection probes: 40 and 80 nm AuNPs (commonly utilized nanoparticles for colorimetric LFIAs), and previously reported PtNCs.^23^ To produce the signal amplifying nanoparticles (SAPs), catalytically active platinum nanoparticles^23^ (Figure S1) were functionalized with rabbit monoclonal secondary antibodies against mouse IgG (ab125913, abcam). SAPs were produced to bind to the detection probes through secondary antibody interactions, forming an aggregate network of nanoparticles. On functionalization of the catalytically active platinum nanoparticles with secondary antibodies, a reduction in catalytic activity was observed (Figure S2), consistent with a reduction in the exposed surface area for catalytic disproportionation of H_2_O_2_. After 30 min, approximately 64% of catalytic activity was retained in the functionalized SAPs.

It was hypothesized that the formation of a coupled nanoparticle aggregate network could produce signal enhancement in nanoparticle-based immunoassays. On forming an aggregate network, the coupled nanoparticle probes could lead to an increased number of immobilized nanoparticles per antigen–antibody binding interaction. Further, owing to the catalytic nature of the SAPs (a peroxidase mimic), signal enhancement could be achieved through addition of a chromogenic substrate (such as TMB). This could be utilized in plate-based assays to produce a colorimetric substrate, or paper-based assays to deposit an insoluble precipitate at the test line (as previously reported by Loynachan et al.^23^), Figure 1.

**Figure 1:**
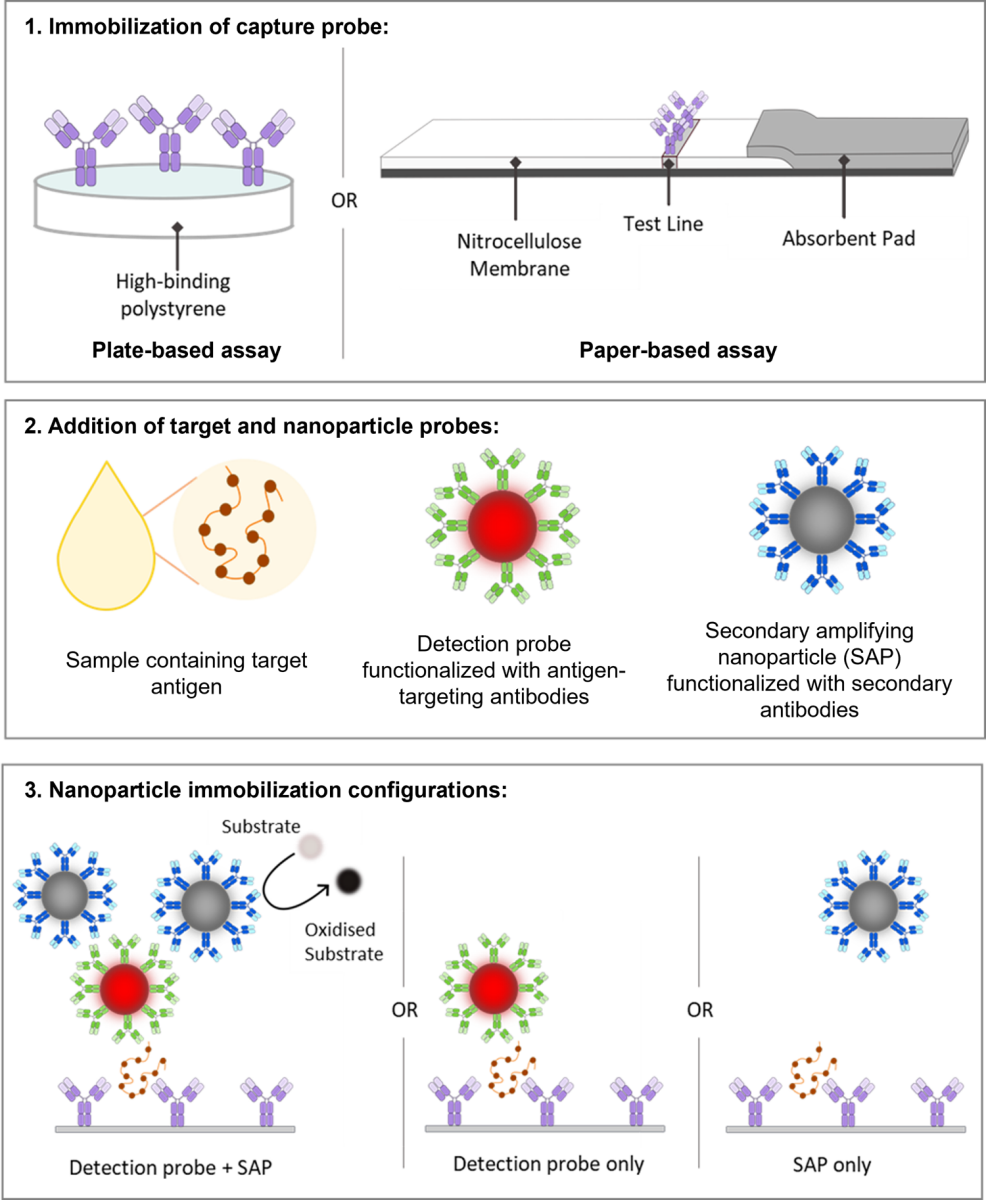
Schematic of the coupling mechanism used to produce signal enhancement in plate- and paper-based immunoassays. (1) Rabbit monoclonal primary antibodies against the SARS-CoV-2 nucleocapsid (N) protein are immobilized onto high-binding polystyrene or nitrocellulose membranes to produce functional capture probes. (2) The sample (containing the antigen) is then incubated with the nanoparticle probes (detection probes and/or SAPs), before interaction with the capture probe. Detection probes consist of nanoparticles functionalized with primary mouse monoclonal antibodies against SARS-CoV-2 N protein target. SAPs consist of catalytically active platinum nanoparticles, functionalized with rabbit monoclonal secondary antibodies against mouse IgG. (3) Illustration of sandwich complex formation on addition of target biomarker and nanoparticle probes to the immobilized capture probe. On addition of the SAPs, enhancement of the signal is achieved by increasing the number of nanoparticles immobilized in the capture region and further enhanced through the use of chromogenic substrates with the catalytically active SAPs.

### Assessment of Coupling Network Formation

To assess the ability of the SAP to couple to the detection probes, transmission electron microscopy (TEM) was used to visualize the network that forms on incubation of detection probes and SAPs. Here, 40 or 80 nm AuNP detection probes were used alongside the SAPs. The use of the PtNC detection probes alongside the SAPs was not imaged here, owing to the two entities having the same size and morphology, rendering them practically indistinguishable via TEM. The detection probes and SAPs were incubated briefly (in a 1:1 particle number ratio), before drop casting and drying in air on a TEM grid (Figure 2A). TEM micrographs illustrate the clear formation of an extended aggregation network in both cases, whereby multiple detection probes are able to form contacts with multiple SAPs (Figure 2B,2C). As a control, bare 40 or 80 nm AuNPs were incubated with bare SAPs. No visible, ordered network was formed in this case, indicating that the network formation was mediated through secondary antibody interactions (Figures S3 and S4).

**Figure 2:**
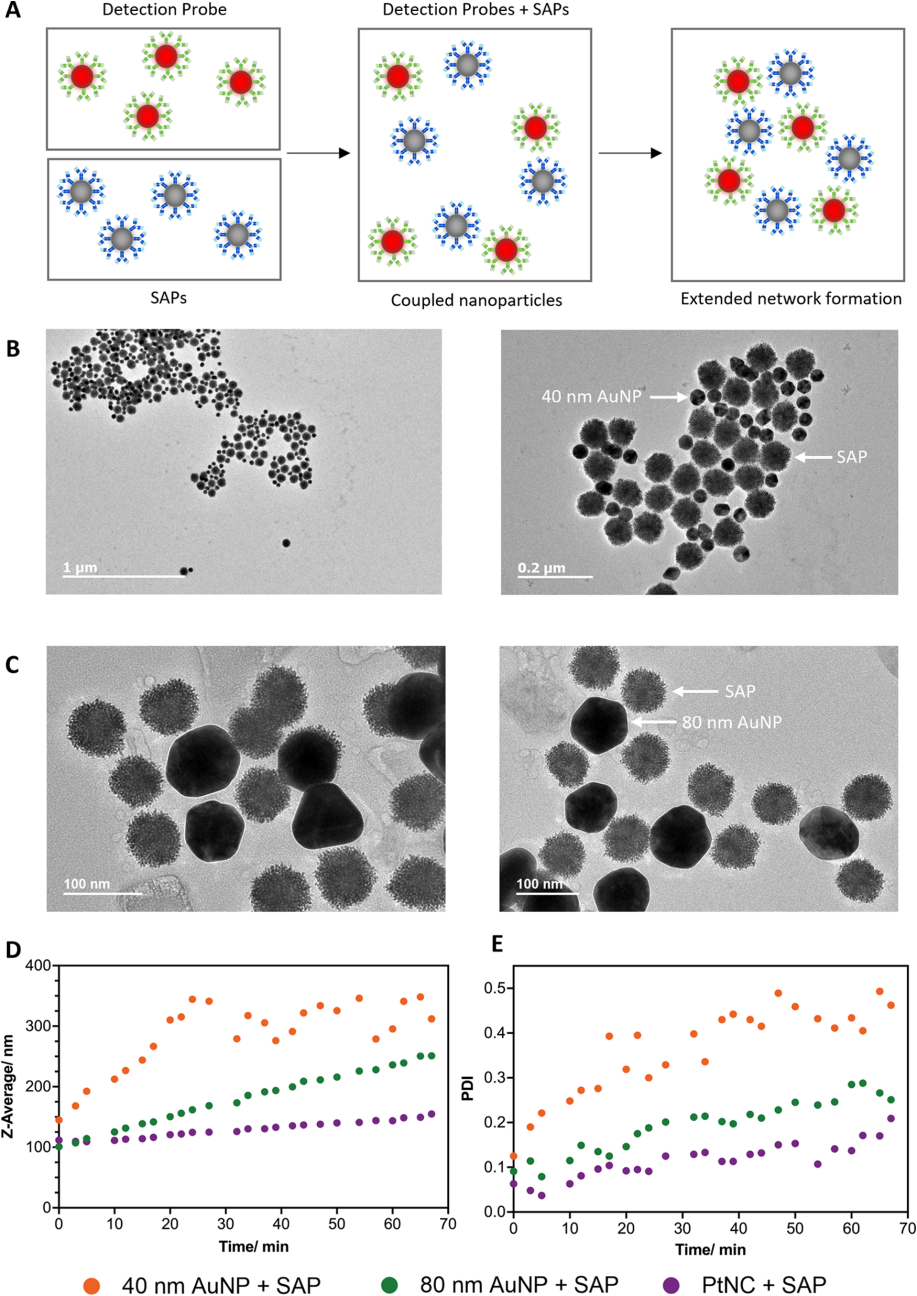
Characterization of the network formation between detection probes and SAPs. (A) Schematic of coupled nanoparticle network formation on incubation of detection probes and SAPs. (B) TEM micrographs of network formation between 40 nm AuNP detection probes and SAPs. (C) TEM micrographs network formation between 80 nm AuNP detection and SAPs. (D) Network formation between detection (40 nm AuNP, 80 nm AuNP, and PtNCs) and SAPs (1:1 particle number ratio), as measured by increase in Z-average through DLS. E: Changes in polydispersity index (PDI) on network formation between detection (40 nm AuNP, 80 nm AuNP, and PtNCs) and SAPs, as determined by DLS.

To probe the level of extended aggregate network formation, dynamic light scattering (DLS) was employed to determine nanoparticle size and polydispersity (PDI) of the coupled nanoparticle network over time. Initially, the hydrodynamic diameter and polydispersity of bare and functionalized nanoparticles (PtNCs, SAPs, 40 nm AuNP, and 80 nm AuNP) was assessed (Figures S5–S7). On functionalization with antibodies, nanoparticle size was shown to increase, while maintaining a narrow size distribution. To assess aggregate network formation, detection probes (PtNCs, 40 or 80 nm AuNPs) and SAPs were mixed in a 1:1 particle number ratio and continuously monitored by DLS over the incubation period (Figure 2D,2E). Over the incubation period, both hydrodynamic diameter and polydispersity of the sample increased, indicating the formation of a coupled aggregate nanoparticle network. This is further exemplified through the intensity-diameter plots, demonstrating a shift in the mean diameter and a broadening of the observed diameter distribution (Figure S8). For the incubation of 40 nm AuNPs with SAPs, an increase in Z-average was observed. However, this trend was less linear than for 80 nm AuNP or PtNCs with SAPs. It is hypothesized that the variable Z-average was due to aggregate clusters, composed of differing numbers of 40 nm AuNPs and SAPs, and the formation of a more extended aggregate network. This was less apparent for 80 nm AuNP or PtNCs with SAPs due to the detection probes having a hydrodynamic diameter close to that of the SAPs. The combination of 40 nm AuNPs and SAPs also exhibited a higher initial PDI, compared to the other nanoparticle conditions, owing to the difference in hydrodynamic diameter between the two nanoparticle species. This is further highlighted by the larger final PDI, which supports the hypothesis that aggregate clusters of different sizes (differing number of nanoparticle species in the cluster) formed. The TEM and DLS data give a strong indication that coupled aggregate networks between detection probes and SAPs can form.

### Application of Coupling Network to Plate-Based Assays

After assessing the network formation between detection probes and SAPs, plate-based assays were utilized to assess the level of signal enhancement that could be achieved. Here, capture antibodies (rabbit monoclonal antibody against SARS-CoV-2 N protein) were immobilized onto the wells of a high-binding polystyrene plates. The species of the capture and detection immunoreagents must be different, so to prevent nonspecific interactions with the SAPs. The N protein antigen was added to the wells and incubated to facilitate interactions between the capture antibody and antigen. The wells were then washed, before addition of nanoparticle probes in a single step before further incubation. For assessment of the generated signal enhancement, detection probes were added with and without SAPs (i.e., detection probe only, detection probes and SAPs, or SAPs only), Figure 3A. The wells were subsequently washed before addition of catalytic substrate solution (TMB and hydrogen peroxide), which would be oxidized by the presence of the catalytic nanoparticles (e.g., SAPs).

**Figure 3:**
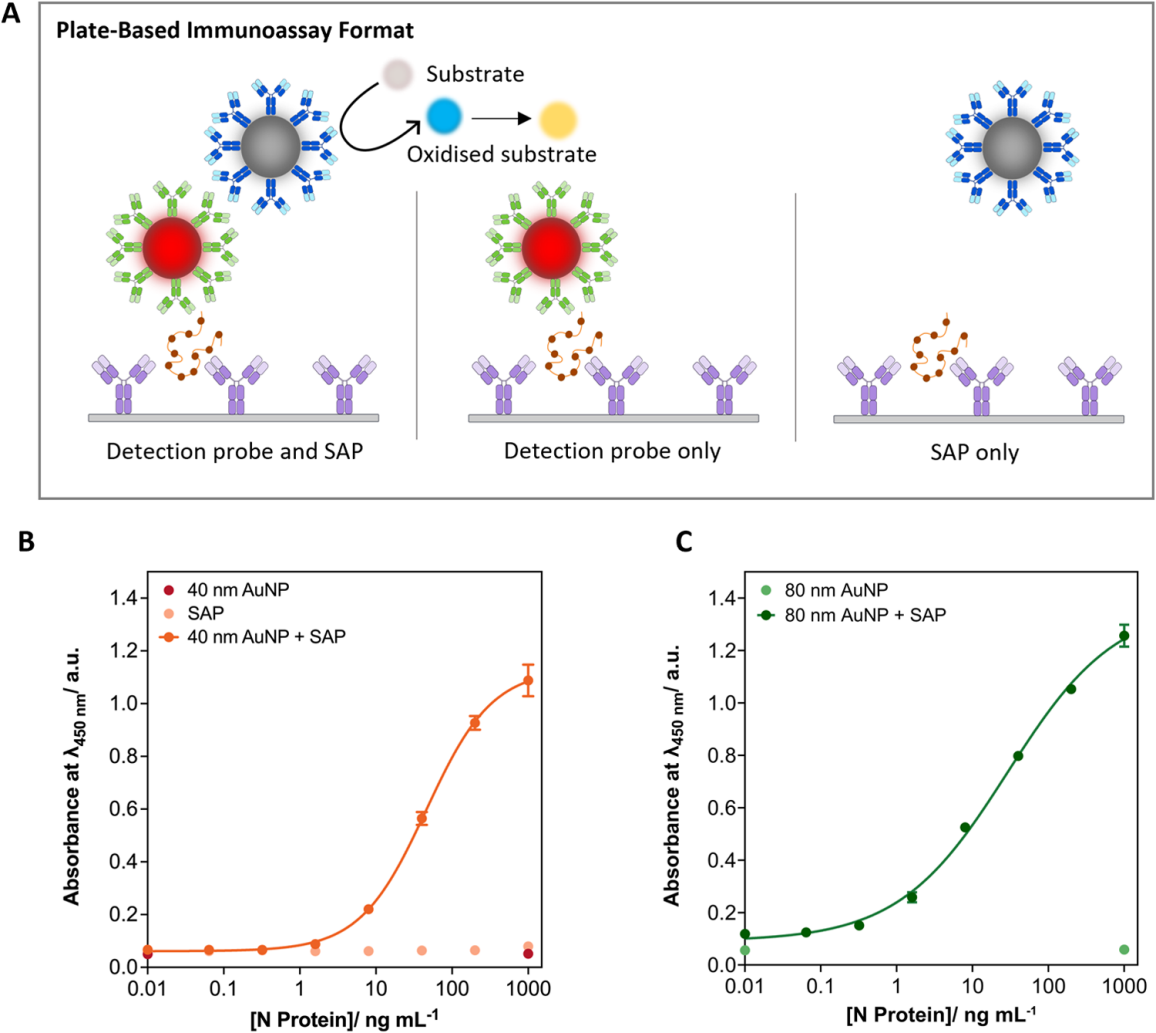
Use of aggregate nanoparticle network formation to generate signal enhancement in plate-based immunoassays assays, utilizing TMB as the chromogenic substrate. (A) Schematic to illustrate the generation of signal enhancement utilizing coupled detection probes and SAPs. (B) Serial dilution of N protein antigen and use with 40 nm AuNP detection probes (1:1 particle number ratio), *n* = 3. Data shown as mean ± SD. (C) Serial dilution of N protein antigen and use with 80 nm AuNP detection probes (1:1 particle number ratio), *n* = 3. Data shown as mean ± SD.

Initially, the plate-based signal enhancement platform was investigated by using 40 and 80 nm AuNP detection probes. As expected, when used in isolation (no addition of SAP), no concentration dependent colorimetric signal was produced for both 40 and 80 nm AuNP detection probes, even at high concentrations of N protein (Figure 3B,3C). When utilized in conjunction with the SAPs (added in a single step, where the detection probes and SAPs were premixed in a 1:1 particle number ratio before addition to the well plate), an antigen concentration dependent colorimetric signal was produced. The colorimetric signal was also not observed on addition of SAPs alone, indicating no cross-reactivity between the capture antibody, antigen and SAPs. The absorbance values (λ_max_ = 450 nm) were plotted as a function of N protein concentration (Figure 3B,3C) to yield a statistically driven limit of detection (LOD) (Table S1). The LOD for the signal enhanced 40 nm AuNP and 80 nm AuNP detection probe was 1.53 ng mL^–1^ (confidence interval (CI) 0.80–2.93 ng mL^–1^) and 0.38 ng mL^–1^ (CI 0.24–0.62 ng mL^–1^), respectively. The LOD for the detection probe only assay could not be extracted due to the lack of concentration dependent signal observed. Therefore, signal enhancement was achieved through the coupling between detection probes and SAPs to form a network of nanoparticles.

### Application of Coupling Network to Paper-Based Assays

To further explore the ability of the coupled aggregate network to produce signal enhancement in immunoassays, we developed a paper-based LFIA to detect the N protein antigen in spiked samples, using the aforementioned detection probes (PtNCs, 40 or 80 nm AuNPs). LFIAs were fabricated in half-dipstick format. In this format, the capture immunoreagent (rabbit monoclonal antibody against SARS-CoV-2 N protein) was striped onto the nitrocellulose membrane to form the test line. The nitrocellulose membrane was fabricated into a half dipstick LFIA through the addition of an absorbent pad. The assay was run by adding the nanoparticle probes (detection probes and/or SAPs) to the spiked sample, before insertion of the LFIA strip directly into the sample (Figure 4A)

**Figure 4:**
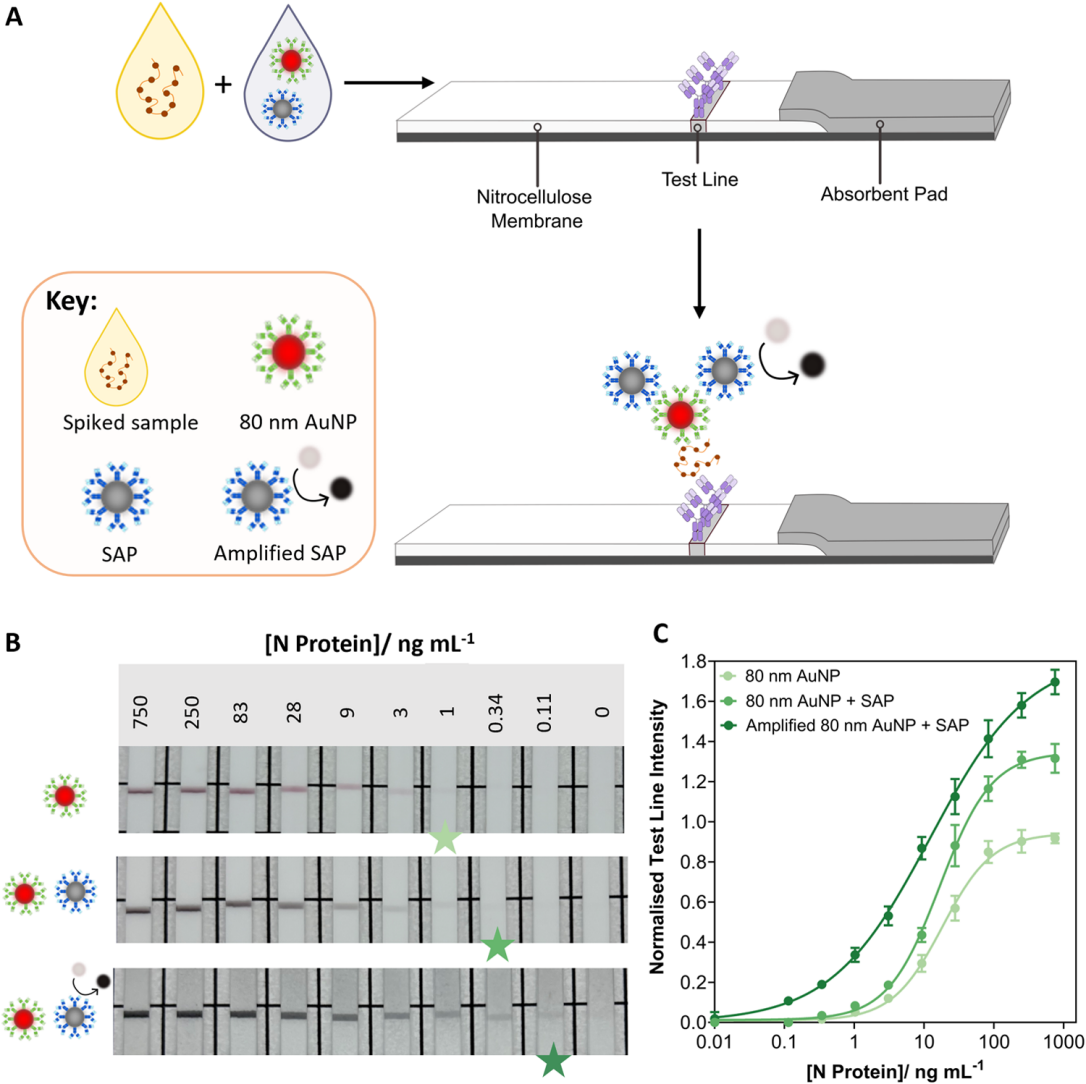
LFIAs utilizing network formation to induce signal enhancement. (A) Schematic of a half dipstick LFIA under the premixed nanoparticle regime. The nanoparticle probes (detection probes and SAPs) are added to the sample, spiked with N protein antigen at varying concentrations. The LFIA strip is then added into the sample. A signal is observed through binding of the antigen-detection probe complex, whereby the SAPs provide signal enhancement through an increased number of nanoparticles immobilized on the test line, and inclusion of catalytically active nanoparticles. (B) Representative images of serial dilution of N protein LFIA strips utilizing 80 nm AuNP detection probes and SAPs (1:6 particle number ratio). Catalytic amplification was utilized to further enhance the signal. The assay was conducted in a spiked buffer sample matrix. The star on each set of LFIA strips represents the visual LOD. (C) Extracted test line pixel intensity for N protein serial dilution with 80 nm AuNP detection probes and SAPs, *n* = 3. Data shown as mean ± SD.

Initially, the signal enhancement platform was performed using spiked running buffer as a sample matrix. As with the plate-based assays, the detection probes and SAPs were premixed before addition to the assay. Here, the 80 nm AuNP detection probe and SAPs were briefly incubated (in a 1:6 particle number ratio), before the solution was allowed to flow up the LFIA strip. By utilizing the 80 nm AuNP detection probe and SAPs in a single step, a black/gray test line was visualized (Figure S9). This indicates the successful coupling between the 80 nm AuNP detection probe and SAP. In contrast, the use of 80 nm AuNP detection probes only produced a distinctive red test line (Figures 4B and S10). A 3-fold visual signal enhancement was observed on addition of the SAP, compared to using 80 nm AuNP detection probes only. The LFIA strip was then immersed in a solution containing catalytic substrate solution (4-chloro-1-naphthol/3,3′-diaminobenzidine tetrahydrochloride (CN/DAB) and hydrogen peroxide), where the substrate was oxidized on the test line to produce an insoluble black precipitate (Figures 4B and S9). The test line intensity was further increased (3-fold visual enhancement) by utilizing the peroxidase mimicking activity of SAPs. This method improved the visual and statistical LODs (calculated from the extracted test line intensities and fitting with a 4-parameter logistic regression, Figure 4B and Tables S2 and S3). The signal enhancement generated *ca*. 4.4-fold improvement in the LOD when comparing the 80 nm AuNP detection probe only and amplified 80 nm AuNP and SAP assays (LOD: 1.68 ng mL^–1^ (CI 0.86 – 3.26 ng mL^–1^) to 0.38 ng mL^–1^ (CI 0.24 – 0.59 ng mL^–1^), Tables S2 and S3). Further, an alternate assay format, utilizing the sequential flow of 80 nm AuNP detection probes and SAPs was investigated (Figure S11). On addition of SAPs, the red 80 nm AuNP test line turned black/gray, indicating successful coupling of the nanoparticle probes. The signal was further enhanced on employment of chromogenic substrate (Figure S10). The same signal enhancement methods were then investigated for use with 40 nm AuNP detection probes with SAPs, using spiked running buffer (Figures S12 and S13). As with 80 nm detection probes, the test line turned from red (40 nm AuNP only) to black/gray on addition of SAPs (after premixing or sequential flow), and the test line intensity increased further on catalytic amplification. The time to result was found to be the same regardless of with or without SAPs, showing that network formation did not significantly perturb the flow rate. This illustrates that the use of SAPs can significantly enhance the signal of AuNP-based LFIAs, a common nanoparticle choice in commercial LFIAs.

To further demonstrate the versatility of this signal amplification platform to operate with a range of detection probes, we utilized the premixed assay format with PtNC detection probes (Figures S14 and S15). The test line intensities were fitted with a 4-parameter logistic regression to extract LOD values. After catalytic enhancement, the LOD for the PtNC detection probe assay was improved *ca*. 2.7-fold (0.38 ng mL^–1^ (CI 0.14 – 1.04 ng mL^–1^) to 0.14 ng mL^–1^ (CI 0.075 – 0.26 ng mL^–1^)) for the premixed PtNC detection probe and SAP assay (Tables S4 and S5). As previously observed, signal enhancement was achieved via the increase in nanoparticle accumulation at the test line (signal increase prior to catalytic amplification), as well as providing additional catalytical enhancement (signal further increases after catalytic enhancement). The maximum signal intensity above the background for the PtNC and SAP assay was lower than that of the PtNC only assay due to the formation of a sharper test line. When the SAP is used in isolation, a weak test line was only observed at a very high antigen concentration (750 ng mL^–1^). However, no test line was observed at lower antigen concentrations.

### Application of Coupling Network with Complex Sample Matrices

The aforementioned LFIA validation utilized spiked buffer samples (containing blocking protein (β-casein) and surfactants (Tween20) in PBS) as the sample matrix. To further assess the ability of the coupled aggregate network to produce signal enhancement, we developed a half-dipstick LFIA using spiked human pooled saliva samples. Detection probes, consisting of 40 nm AuNPs, were utilized in the study owing to their ubiquitous use in LFIAs. The premixed format was utilized (with a 1:3 particle number ratio of 40 nm AuNP detection probes to SAPs), demonstrating signal enhancement on addition of SAPs, both prior to and postcatalytical amplification (Figures 5A,5B and S16). On extraction of test line intensities and fitting with a 4-parameter logistic regression, the LOD values were improved by *ca*. 40-fold, from 9.38 ng mL^–1^ (3.76–23.4 ng mL^–1^, 40 nm AuNP only) to 0.22 ng mL^–1^ (0.033–1.38 ng mL^–1^, amplified premixed 40 nm AuNP and SAPs) as shown in Table S6 and S7. The visual LOD was found to be comparable to using the assay with spiked buffer samples (Figure S12), indicating minimal interference from the presence of the saliva matrix.

**Figure 5:**
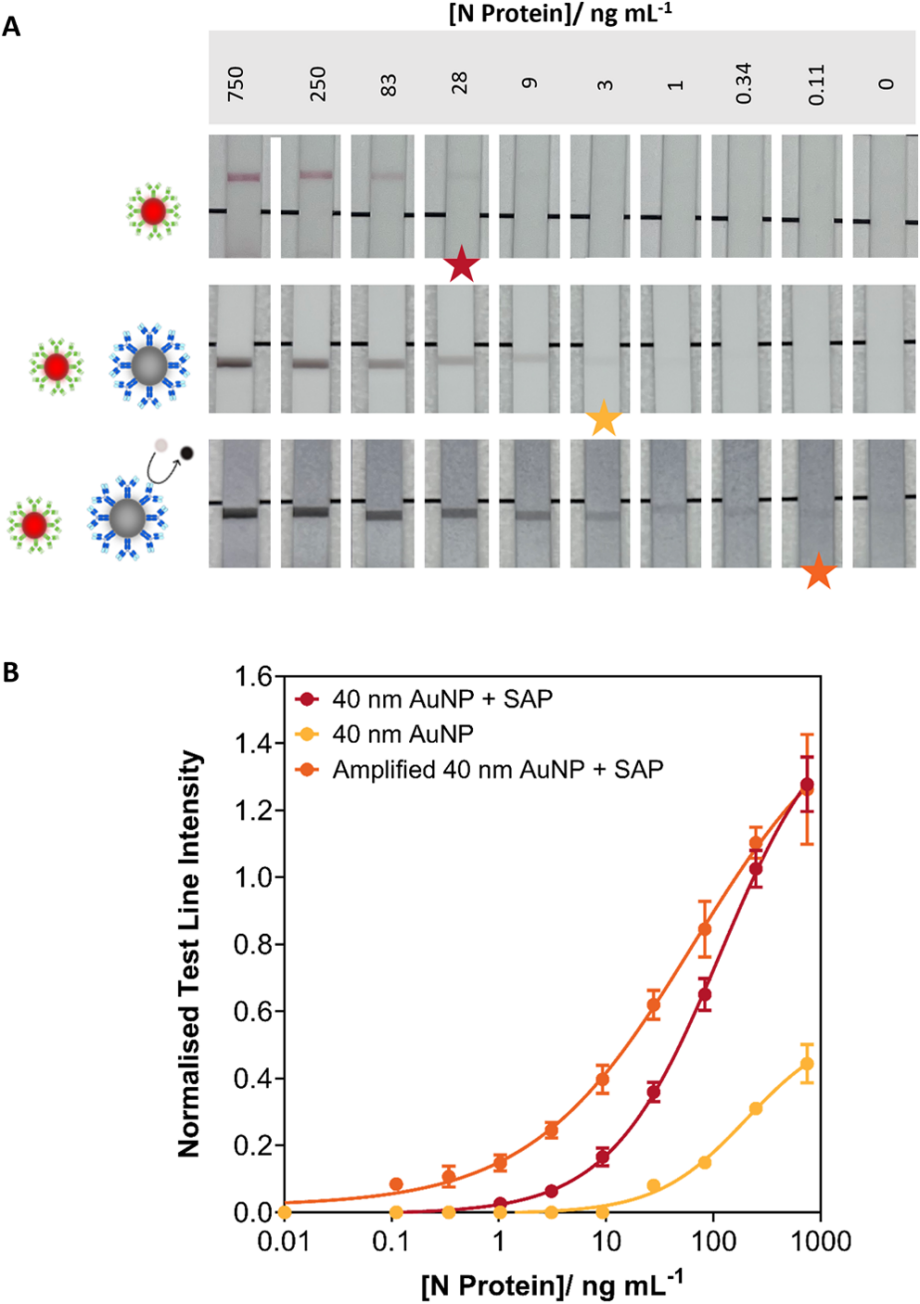
Half dipstick LFIA utilizing network formation to induce signal enhancement in spiked human saliva. **(A)** Representative images of serial dilution of N protein LFIA strips utilizing premixed 40 nm AuNP detection probes and SAPs. Catalytic amplification was utilized to further enhance the signal. The assay was conducted in a spiked human pooled saliva sample matrix. The star on each set of LFIA strips represents the visual LOD. **(B)** Extracted test line pixel intensity for N protein serial dilution with 40 nm AuNP detection probes and SAPs (1:3 particle number ratio) in spiked human pooled saliva, *n* = 3. Data shown as mean ± SD.

The N protein concentration at the statistical LOD can be correlated with genome equivalents mL^–1^ (GEs mL^–1^), allowing for comparison to commercially available rapid diagnostic tests (RDTs).^31,32^ The statistical LOD of the signal enhanced assay (0.22 ng mL^–1^) can be estimated as 5 × 10^4^ genome GEs mL^–1^.^31^ The WHO established a predefined performance criterion of RDTs, with an acceptable analytical LOD of approximately 10^6^ GEs mL^–1^, and a desirable LOD of 10^4^ GEs mL^–1^.^32,33^ Reports have investigated the LOD of commercially available RDTs to detect SARS-CoV-2, with a number of commercially available LFIAs achieving LODs of approximately 10^5^ GEs mL^–1^.^32^ The LOD reported in this work demonstrates an improved sensitivity compared to a number of existing commercially available LFIAs. We anticipate performing more work to assess the clinical sensitivity of the signal enhancement platform in future studies.

The signal enhancement platform was further demonstrated by using the sequential flow assay format (Figures S11, S17 and S18). It should be noted that for the 40 nm AuNP detection probe assay, the level of visual signal enhancement achieved in the premixed format was greater than for 80 nm AuNP or PtNC detection probes. It is hypothesized that this originates from the more extended network formation between 40 nm AuNP detection probes and SAPs, as illustrated in the DLS and TEM characterization (Figure 2B, 2D, and 2E). This is further highlighted as the premixed assay format produced more significant signal enhancement than the sequential flow format, owing to the more extended interaction time between the 40 nm AuNP detection probes and SAPs. This work was designed to show that the catalytic signal enhancement is maintained in complex sample matrix. A thorough clinical validation would be required to assess its use as a clinical *in vitro* diagnostic assay, which was deemed beyond the scope of this work.

## Conclusions

Here, we demonstrated a signal enhancement platform that utilizes secondary antibody interactions to generate an aggregation network between detection probes (with antigen targeting ability) and SAPs (with intrinsic catalytic activity). This platform is compatible with a range of detection probe identities, including AuNPs and platinum nanoparticles. Initially, we characterized the extent of aggregate network formation, utilizing TEM to visualize network formation, and DLS to probe network formation as a function of time. Functionally, we showcased the signal enhancement platform in plate-based assays, where the use 40 and 80 nm AuNP detection probes alongside SAP detection probes produced a concentration dependent signal. As well as providing signal enhancements in plate-based assays, we hypothesize that this platform could be utilized for the high-throughput screening of affinity agent pairs. In particular, the screening of functionalized detection probes, where an immunoreagent is functionalized to a nanoparticle which is not intrinsically catalytically active, for the optimization of LFIAs. Currently, matched antibody pairs are typically screened using enzyme-linked immunosorbent assays (ELISAs), surface plasmon resonance (SPR) or biolayer interferometry (BLI), whereby detection antibodies are utilized while mobile in solution.^5^ However, in LFIA format, detection antibodies are coupled to nanoparticle probes, leading to high local concentrations of antibodies around the detection probe. This platform provides a workflow for using noncatalytically active nanoparticles in ELISA-like assays, whereby a secondary catalytic nanoparticle provides the signal generation.

Furthermore, we demonstrated that signal enhancement can also be achieved on the test line of LFIAs. This is achieved via two mechanisms: by increasing nanoparticle accumulation, and the incorporation of nanoparticle catalytic activity to amplify the generated signal. This platform functions effectively in both clean (buffer) and complex sample (human pooled saliva) matrices, producing comparable levels of signal amplification. In spiked human pooled saliva, the use of SAPs produced a statistically significant decrease in the LOD, demonstrating a 40-fold improvement in LOD. Additionally, the signal enhancement strategy can be readily integrated into standard LFIA architecture, without the need for complex operation steps or instrumentation to interpret test line intensity. As such, we propose that this signal enhancement strategy could readily be integrated into LFIAs suffering from inadequate clinical sensitivity.

## Experimental Section

### Immunoreagent Materials

Two antigen-targeting commercially available monoclonal antibodies were utilized in the assay: N9547 (Meridian Bioscience, supplied by Biorbyt) as the detection antibody, and 40143-R001 (SinoBiological) as the capture antibody. Rabbit monoclonal secondary antibody against mouse IgG was purchased from abcam (ab125913). Antigens were purchased from SinoBiological (catalogue numbers: 40588-V08B).

### Synthesis of Platinum Nanocatalysts

As described by Loynachan et al.,^23^ 15 nm AuNP seeds were synthesized by the reduction of HAuCl_4_ by sodium citrate. Briefly, 10 mL of gold­(III) chloride trihydrate aqueous solution (20 mM, Sigma) was added to 180 mL of Ultra Pure Distilled Water (UPDW, Invitrogen) under reflux at 100 °C. The temperature was then reduced to 70 °C, and the reaction initiated by fast injection of 10 mL of trisodium citrate dihydrate (68 mM, Sigma) with vigorous stirring and refluxed for 5 min. The resulting *ca*. 15 nm gold nanoparticle (AuNP) seeds were cooled to RT and subsequently stored at 4 °C.

Platinum nanocatalysts with a hydrodynamic diameter of *ca*. 90 nm were synthesized via the reduction of chloroplatinic acid hydrate on to the 15 nm AuNP seeds. Glassware (24 mL glass vial) was washed 3x with 10 mL of UPDW before beginning the synthesis protocol. In a typical synthesis, 620 μL of 15 nm AuNP seed (10 nM) was added to a glass vial containing 19.4 mL of UPDW, followed by the addition of 400 μL of 20 w/v% of poly­(vinylpyrrolidone) (PVP, MW 10 kDa, Sigma). The solution was briefly vortexed and incubated without stirring for 5 min. To the polymer coated AuNP seed solution, 800 μL of l-ascorbic acid (100 mg mL^–1^, Sigma) was added, followed by the addition of 800 μL of chloroplatinic acid hydrate (100 mM, Sigma). The resulting solution was briefly vortexed and immediately incubated at 65 °C for 45 min until the color of the solution changed from red to black. Platinum nanocatalysts were then cooled to RT, and excess reagents removed through three sequential washing cycles at 7000 rcf for 5 min with resuspension into UPDW. After the final wash step, platinum nanocatalysts were resuspended in UPDW and stored at 4 °C.

### Production of PtNC Detection Probes

PtNCs were functionalized with antibodies via physisorption, following previously reported protocols.^35,23^ Briefly, to a Protein LoBind Eppendorf, 100 μL PtNC solution (600 pM) was added, before the addition of 10 μL of conjugation buffer (100 mM HEPES, pH 6.5). Antibody solution (mouse monoclonal antibody against SARS-CoV-2 N protein, N9547) was added at a ratio of 1:125 equiv of PtNC:antibody. The solution was incubated at room temperature (RT) under shaking (700 rpm) for 3 h. 100 μL of blocking buffer (2 wt % β-casein in Dulbecco’s PBS (DPBS)) was then added, and the solution incubated at RT under shaking (700 rpm) for 1 h. The resultant conjugation was then washed 3× via centrifugation (1300 rcf for 10 min) and replacement of the supernatant with running buffer (0.2 wt % β-casein, 0.2 wt % Tween20 in DPBS). The final solution was made up to a final volume of 100 μL (600 pM).

### Production of 40 nm AuNP Detection Probes

AuNPs (40 nm, BBI) were functionalized with antibodies via physisorption.^34^ Briefly, to a 1.75 mL glass vial, 300 μL AuNP solution (OD@530 = 1, 105 pM) was added, before the addition of 60 μL of conjugation buffer (100 mM HEPES, pH 7). Antibody solution (mouse monoclonal antibody against SARS-CoV-2 N protein, N9547) was added at a ratio (AuNP:antibody) of 1:400 equiv. The solution was incubated at RT without shaking for 3 h. After this time, 30 μL of blocking buffer (2 wt % β-casein in 20 mM carbonate buffer, pH 9.8) was then added, and the solution incubated at RT without shaking for 1 h. The resultant conjugation was then washed 3x via centrifugation (5000 rcf for 10 min) and replacement of the supernatant with running buffer (0.2 wt % Tween20 in 20 mM HEPES, pH 7). The final solution was made up to a final volume 300 μL (OD@530 = 1, 105 pM).

### Production of 80 nm AuNP Detection Probes

AuNPs (80 nm, BBI) were functionalized with antibodies via physisorption.^34,35^ Briefly, to a 1.75 mL glass vial, 300 μL AuNP solution (OD@530 = 5, 51.5 pM) was added, before the addition of 60 μL of conjugation buffer (100 mM HEPES, pH 8). Antibody solution (mouse monoclonal antibody against SARS-CoV-2 N protein, N9547) was added at a ratio (AuNP:antibody) of 1:800 equiv. The solution was incubated at RT under shaking for 3 h (700 rpm). Thirty μL of blocking buffer (2 wt % β-casein in 20 mM carbonate buffer, pH 9.8) was then added, and the solution incubated at RT under shaking (700 rpm) for 1 h. The resultant conjugation was then washed 3 x via centrifugation (5000 rcf for 10 min) and replacement of the supernatant with running buffer (0.2 wt % Tween20 in 20 mM HEPES, pH 8). The final solution was made up to 300 μL (OD@530 = 5, 51.5 pM).

### Production of Signal Amplifying Nanoparticles (SAPs)

PtNCs were functionalized with antibodies via physisorption, following previously reported protocols,^23^ to produce signal amplifying nanoparticles (SAPS). Briefly, to a Protein LoBind Eppendorf, 100 μL PtNC solution (600 pM) was added, before the addition of 10 μL of conjugation buffer (100 mM HEPES, pH 6.5). Antibody solution (rabbit monoclonal antibody against mouse IgG, ab125913) was added at a ratio of 1:125 equiv of PtNC:antibody. The solution was incubated at RT under shaking (700 rpm) for 3 h. 100 μL of blocking buffer (2 wt % β-casein in Dulbecco’s PBS (DPBS)) was then added, and the solution incubated at RT under shaking (700 rpm) for 1 h. The resultant conjugation was then washed 3x via centrifugation (1300 rcf for 10 min) and replacement of the supernatant with running buffer (0.2 wt % β-casein, 0.2 wt % Tween20 in DPBS). The final solution was made up to a final volume of 100 μL (600 pM).

### UV–vis Spectroscopy

Absorption of AuNPs and AuNP conjugates was measured using UV–vis spectroscopy. 80 μL of sample was added to a cuvette, before measuring the absorption spectra and absorption value at 530 nm (NanoDrop 2000c, Thermo Scientific).

### Dynamic Light Scatter

Dynamic light scattering measurements were performed on a Zetasizer Nano ZS (Malvern Instruments, Ltd.) equipped with a 633 nm He–Ne laser. Measurement parameters were set using the Zetasizer Nano software v8.02, and samples equilibrated to RT before measurements.

For investigation of size distribution and polydispersity of the coupled aggregate network, a sample containing detection probes and SAPs was made and measurements continuously taken over 60 min. The detection probes and SAPs were both prepared to a final concentration of 5 pM in 100 μL of DPBS (1:1 particle number ratio).

### Transmission Electron Microscopy

Sample preparation for TEM characterization was performed by diluting nanoparticle probes in UPDW. For characterization of bare catalytically active platinum nanoparticles, a concentration of 100 pM was used. 2.5 μL of sample was drop-casted onto an ultrathin carbon support film on lacey carbon grids (Agar Scientific) and left to air-dry for 30 min.

For characterization of 40 or 80 nm AuNP detection probe and SAPs, a concentration of 20 pM per species was utilized (1:1 particle number ratio). 2.5 μL of sample was drop-casted onto an ultrathin carbon support film on lacey carbon grids (Agar Scientific) and left to air-dry for 30 min. The grid was blotted with 2.5 μL of UPDW to remove excess salt.

TEM imaging was performed using a JEOL 2100F and JEOL 2100Plus operating at 200 kV, equipped with a Gatan Orius SC1000 camera.

### Plate-Based Immunoassays

To the appropriate wells of a 96-well clear flat bottom polystyrene high bind microplate (Corning), 100 μL of capture immunoreagent (rabbit monoclonal antibody against SARS-CoV-2 N protein, 40143-R001, 0.5 μg mL^–1^) was added and incubated overnight at 4 °C. The plate was subsequently washed (3×) with DPBST (DPSB + 0.05 v/v% Tween20). All wells were blocked through the addition of 150 μL of 3 w/v% BSA in DPBS and incubated at RT for 1 h. The wells were subsequently washed (3×) with DPBST. 100 μL of antigen-spiked PBST (SARS-CoV-2 N protein, 40588-V08B, varying concentrations) was then added to the appropriate wells and incubated at RT for 30 min, before wells were then washed (3×) with DPBST. 100 μL of nanoparticle solution (containing: detection probe (40 or 80 nm AuNP), detection probe and SAP, or SAP only. Each nanoparticle species added at a concentration of 2 pM) was added to the appropriate wells and incubated at RT for 30 min. The wells were washed (3×) with DPBST before the addition of 100 μL of freshly prepared TMB solution (1.6 v/v% TMB (0.6 mg mL^–1^) (Sigma) in DMSO (Sigma) + 2 v/v% H_2_O_2_ (Sigma) in 50 mM citrate buffer, pH 5.0). The plate was shielded from light and incubated at RT for 30 min. After exactly 30 min, the reaction was stopped through the addition of 50 μL of H_2_SO_4_ (4 M) to each well. The OD at 450 nm was recorded using a SpectraMax M5 microplate reader (Molecular Devices) and analyzed using GraphPad Prism 9.5.1 (528).

### Fabrication of LFIAs

To produce LFIA strips with specific capture antibodies, an automated liquid dispensing system (BioDot System AD3220) was used. Here, the capture immunoreagent (40143-R001, 1 mg mL^–1^) was filtered through a 0.22 μm filter. The capture immunoreagent was dispensed at a height of 10 mm from the base of the nitrocellulose membrane (CN95 Unisart Nitrocellulose Membrane, Sartorius) before drying for 4 h at 37 °C. Half-dipstick lateral flow strips were assembled onto backing card (KN-PS1060.18, Kenosha) with an overlapping absorbent pad (KN-222-20.1, Ahlstrom-munksjo). Half-dipstick strips were then cut into individual strips with a width of 3 mm using an automated guillotine (BioDot CM5000 Guillotine Cutting System). Half-dipstick strips were stored in a desiccant cabinet before use.

### PtNC Detection Probe LFIA

All LFIA strips were blocked with a solution of 0.5 wt % skimmed milk in DPBS before use. Briefly, fabricated LFIA strips were immersed in 1 mL of 0.5 wt % skimmed milk in DPBS for 15 min. Strips were then washed 3x by immersing the strip in 500 μL of 5 mM Na_2_HPO_4_ pH 7 + 0.01 wt % SDS for 15 min. Strips were dried at 37 °C for 4 h.

PtNC detection probe LFIAs were performed by immersing a constructed LFIA test strip (40143-R001 capture probe, 1 mg mL^–1^) into a 96-well clear flat bottom polystyrene nonbinding surface microplate (Corning) containing. Each well contained: 50 μL sample (N protein spiked into DPBST) and 15 μL of PtNC detection probe (50 pM). When the solution had fully wicked up the LFIA strip (*ca*. 10 min), the strip was transferred into a well containing 100 μL of running buffer (0.2 w/v% β-casein, 0.2 v/v% Tween20 in DPBS) for 10 min. Next, the strip was transferred to an amber Eppendorf containing 500 μL of amplification solution (Pierce CN/DAB Substrate Kit (Thermo Fisher Scientific), adjusted with hydrogen peroxide solution 30 w/w% (Sigma) to a final added peroxide concentration of 4 M) for 10 min. Finally, the strip was moved into an Eppendorf containing 1 mL of Milli-Q water and briefly agitated to stop the reaction. LFIA strips were left to dry for 20 min before being photographed with an iPhone 13 camera and images processed using ImageJ. Extracted pixel intensities were fit with a 4-parameter logistic regression and analyzed using GraphPad Prism 9.5.1 (528) and Detection Limit Fitting software.^36–38^

### PtNC Detection Probe and SAP LFIA

The PtNC detection probe and SAP LFIA was performed according to the above protocol with slight modifications. On addition of nanoparticle probes, 15 μL of PtNC detection probe (50 pM) and of 15 μL SAP (50 pM) was utilized.

### Pre-Mixed AuNP Detection Probe and SAP LFIA

The premixed AuNP detection probe and SAP LFIA was performed according to the above protocol with slight modifications. LFIA strips were utilized without blocking. The sample consisted of 50 μL of N protein spiked running buffer. Detection probe was added to the well (5 μL of 40 nm AuNP (OD@530 = 1, 105 pM) or 5 μL of 80 nm AuNP (OD@530 = 5, 51.5 pM)), followed by addition of the SAP (2.5 μL, 600 pM), before addition of LFIA strip. The solution was allowed to flow up the strip for 10 min. The LFIA strip was then moved to a well containing 50 μL of running and the solution was allowed to flow up the strip for 10 min. The strip was then moved to an Eppendorf containing amplification solution, as outlined above.

### Sequential Flow AuNP Detection Probe and SAP LFIA

The sequential flow AuNP detection probe and SAP LFIA was performed according to the above protocol with slight modifications. LFIA strips were utilized without blocking. The sample consisted of 50 μL of N protein spiked running buffer. Detection probe was added to the well (20 μL of 40 nm AuNP (OD@530 = 1, 105 pM) or 5 μL of 80 nm AuNP (OD@530 = 5, 51.5 pM)), before addition of LFIA strip. The solution was allowed to flow up the strip for 10 min. The LFIA strip was then moved to a well containing 50 μL of running buffer and 2.5 μL of SAP (600 pM), and the solution was allowed to flow up the strip for 10 min. The strip was then moved to an Eppendorf containing amplification solution, as outlined above.

### AuNP Detection Probe LFIA

The AuNP detection probe only LFIA was performed according to the above protocol with slight modifications. LFIA strips were utilized without blocking. The sample consisted of 50 μL of N protein spiked running buffer. Detection probe was added to the well (10 μL of 40 nm AuNP (OD@530 = 1, 105 pM) or 5 μL of 80 nm AuNP (OD@530 = 5, 51.5 pM)), before addition of LFIA strip. The solution was allowed to flow up the strip for 10 min. The LFIA strip was then moved to a well containing 50 μL of running buffer, and the solution was allowed to flow up the strip for 10 min.

### Pre-Mixed 40 nm AuNP Detection Probe and SAP LFIA in Spiked Saliva

The coupled AuNP detection probe and SAP LFIA was performed according to the above protocol with slight modifications. LFIA strips were utilized without blocking. The sample consisted of 50 μL of N protein spiked human pooled saliva (≥3 healthy donors, bulk volumes, Lee Biosolutions). Concentrations stated represent the final N protein concentration, after dilution of spiked human pooled saliva with running buffer (3:2 ratio).

The 5 μL of 40 nm AuNP detection probe (OD@530 = 1, 105 pM) and 2.5 μL of SAP (600 pM) was added to a well containing the sample. The LFIA strip was added and the solution allowed to flow up the strip for 10 min. The LFIA strip was then moved to a well containing 50 μL of running buffer and allowed to flow up the strip for 10 min. The strip was then moved to an Eppendorf containing amplification solution, as outlined above.

### ImageJ Processing

Images of LFIA strips were captured using an iPhone 13 camera. Images imported into ImageJ (ImageJ 1.53k) and converted to gray scale (8-bit) images. A region of interest around the test line was selected (width to height ratio 1:2.5), and peak area pixel density was calculated using the ImageJ software. Test line intensities were normalized to a reference point (grid line) in the image to account for any variation in image lighting.

## Supplementary Material



## Notes

Additional experimental data including characterization of nanoparticle conjugates, serial dilution of LFIA images, LOD analysis, and statistical analysis of extracted LODs (PDF)
