# Supplementary material for: Signal Enhancement in Immunoassays via Coupling to Catalytic Nanoparticles
Source: ACS Sens. 2025 May 20;10(6):4622–33. doi: 10.1021/acssensors.5c00995 (PMC12210247; doi:10.1021/acssensors.5c00995)
Supplement: Supplementary file 1 [file se5c00995_si_001.pdf]

Supporting Information

Signal Enhancement in Immunoassays *via* Coupling to Catalytic Nanoparticles

Christy J. Sadler[1,2], Jan P. Sandler[1], André Shamsabadi[1,2], Leah C. Frenette[1], Adam Creamer*[1,2], Molly M. Stevens*[1,2]

[1]Department of Materials, Department of Bioengineering, Institute of Biomedical Engineering Imperial College London, London, SW7 2AZ, UK

[2]Department of Physiology, Anatomy and Genetics, Department of Engineering Science, Kavli Institute for Nanoscience Discovery, University of Oxford, OX1 3QU, UK

*Corresponding authors: Adam Creamer (adam.creamer@dpag.ox.ac.uk) and Molly M. Stevens (molly.stevens@dpag.ox.ac.uk)

Contents:

Supporting Information Figures: *p02*

Supporting Information Experimental Section: *p21*

References: *p23*

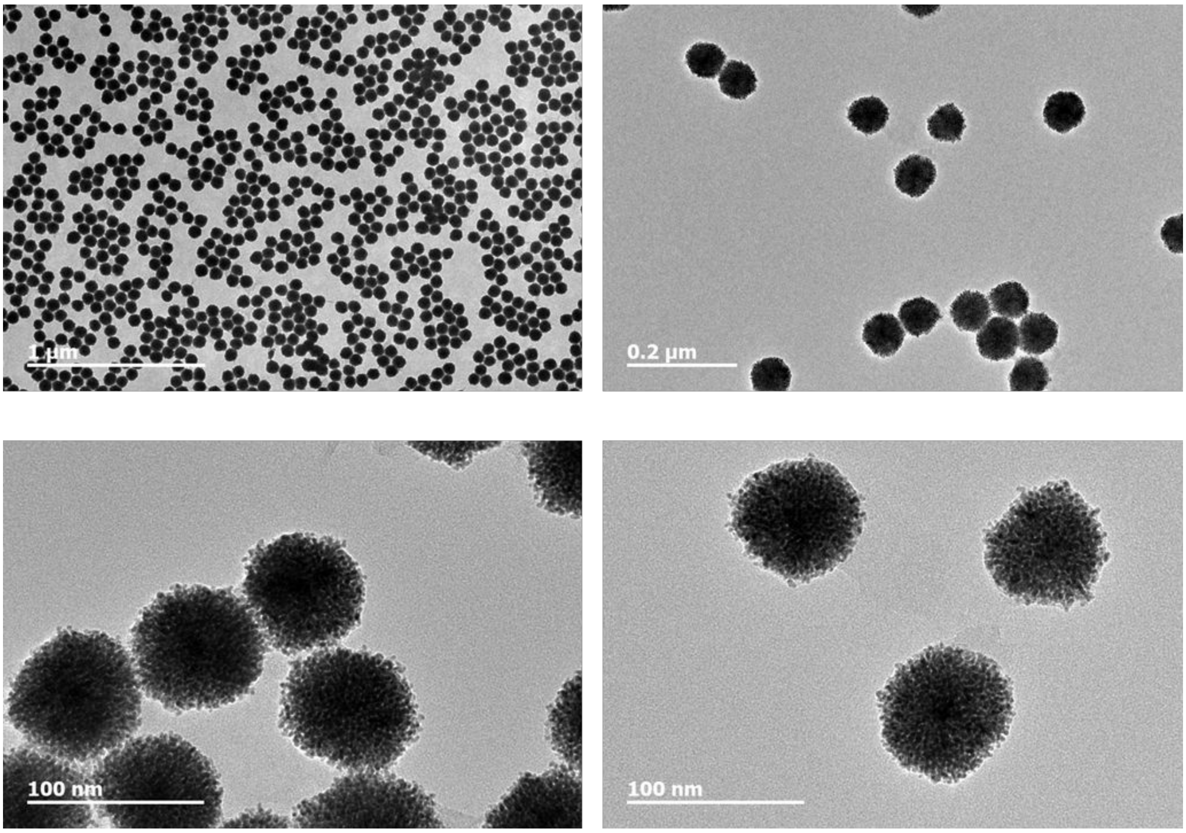

Figure S1: TEM micrographs of catalytically active platinum nanoparticles, the base particles for the production of PtNC detection probes and signal amplifying nanoparticles (SAPs).

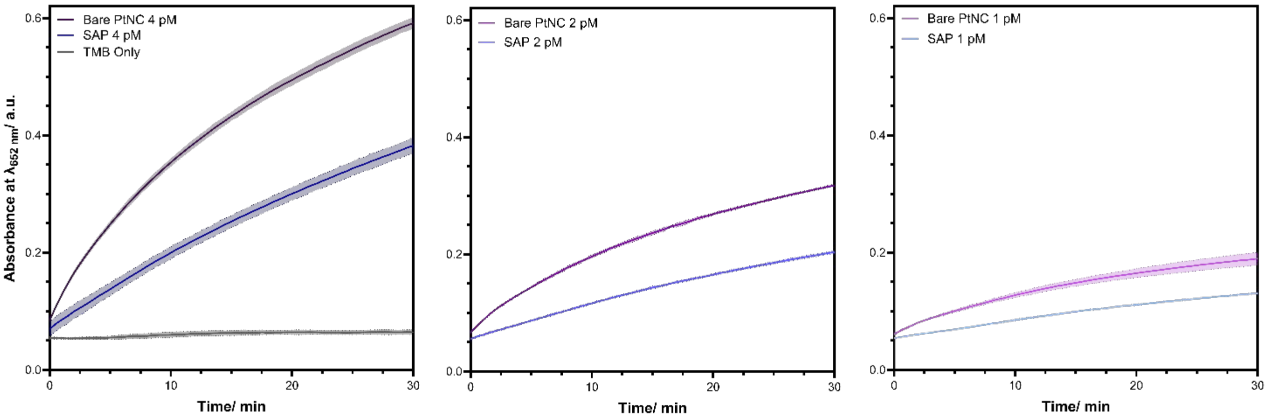

Figure S2: Assessment of catalytic activity of bare catalytically active platinum nanoparticles and SAPs using 1-Step TMB Ultra substrate solution. Absorbance measured at 652 nm as a function of time. Three concentrations of nanoparticles (4, 2 and 1 pM) and TMB only as control are assessed. n = 3.

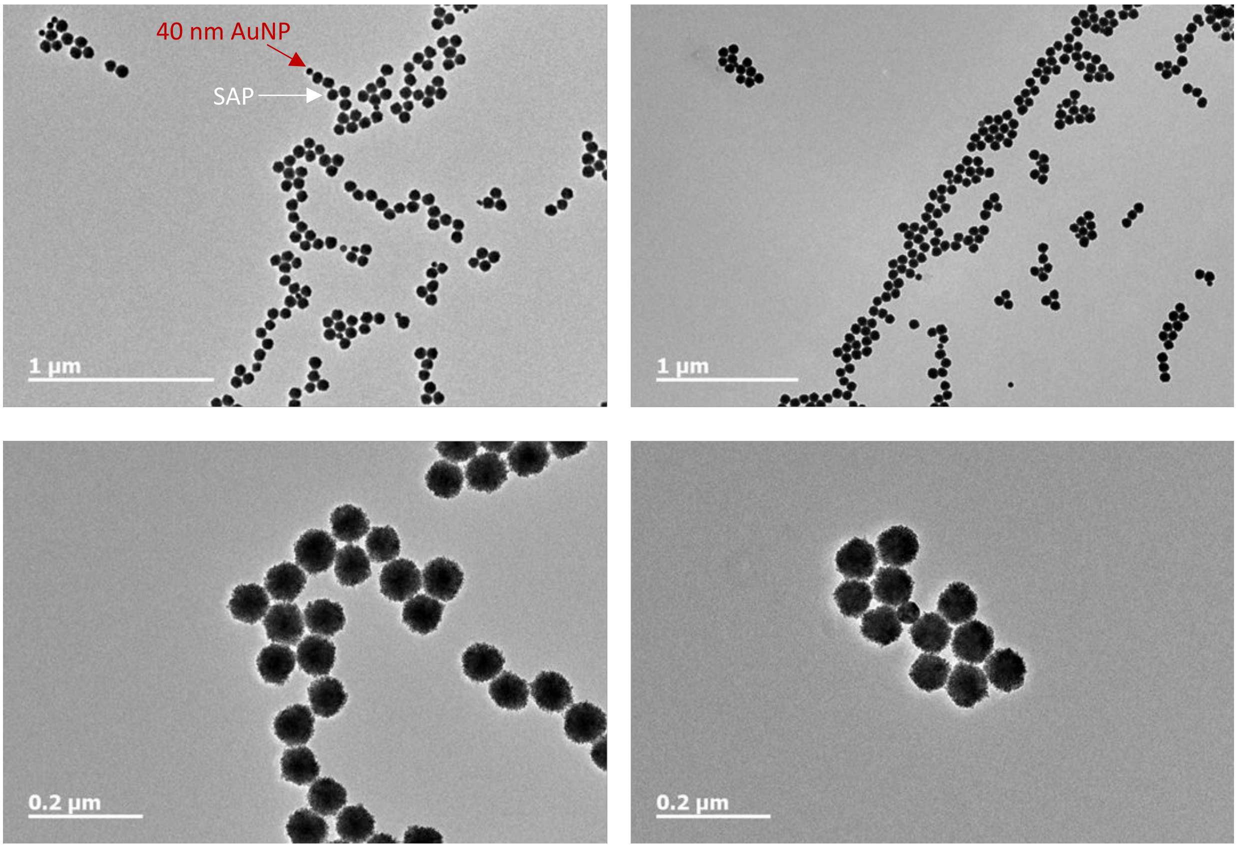

Figure S3: TEM micrographs of bare 40 nm AuNPs and bare SAPs (1:1 particle number ratio), illustrating the absence of network formation when bare nanoparticles are incubated together.

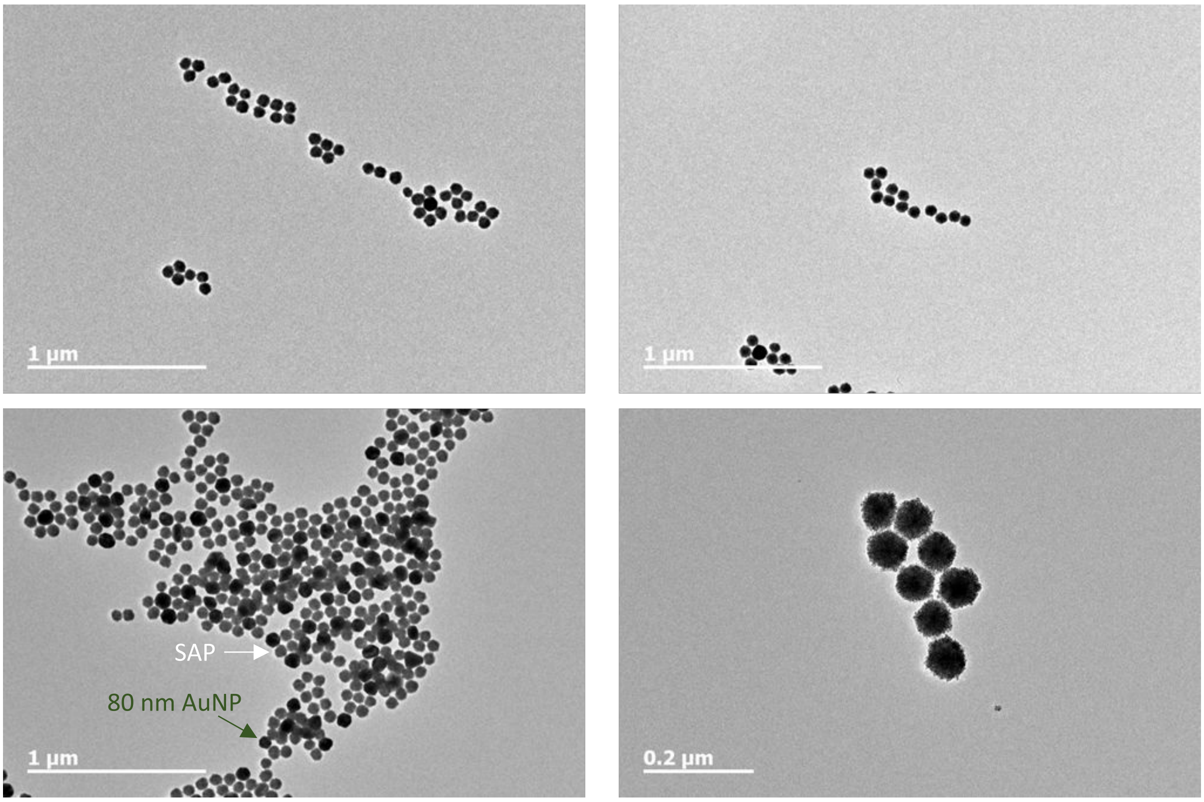

Figure S4: TEM micrographs of bare 80 nm AuNPs and bare SAPs (1:1 particle number ratio), illustrating the absence of ordered network formation when bare nanoparticles are incubated together.

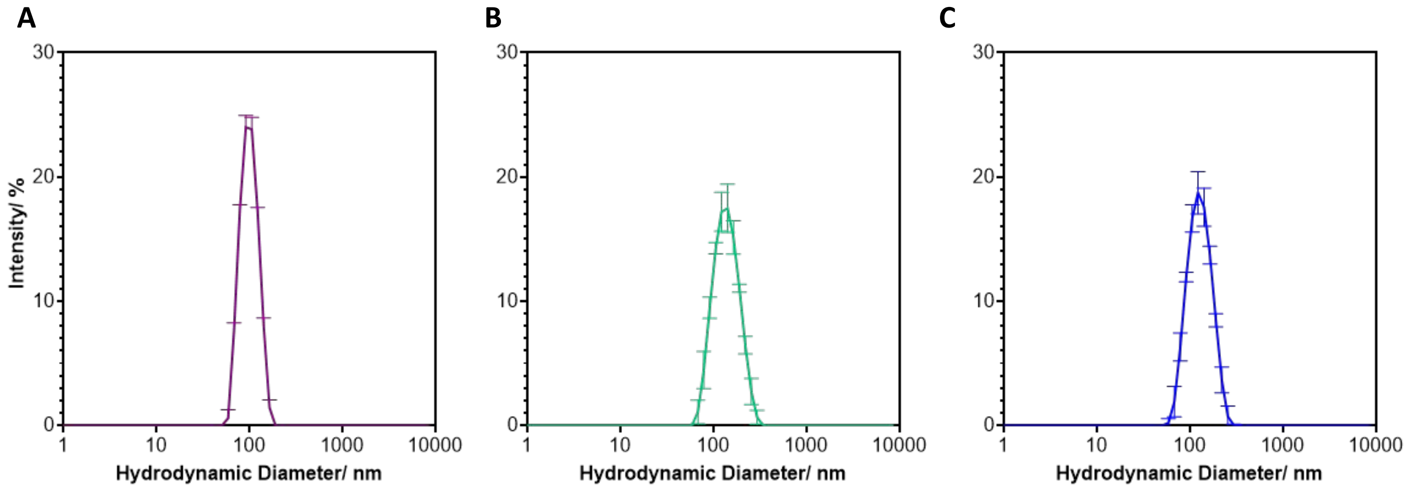

Figure S5: Dynamic light scattering (DLS) of catalytically active platinum nanoparticles. **A:** DLS of bare catalytically active platinum nanoparticles, n = 3. **B:** DLS of PtNC detection probes, n = 3. **C:** DLS of SAPs, n = 3. Data shown as mean ± S.D.

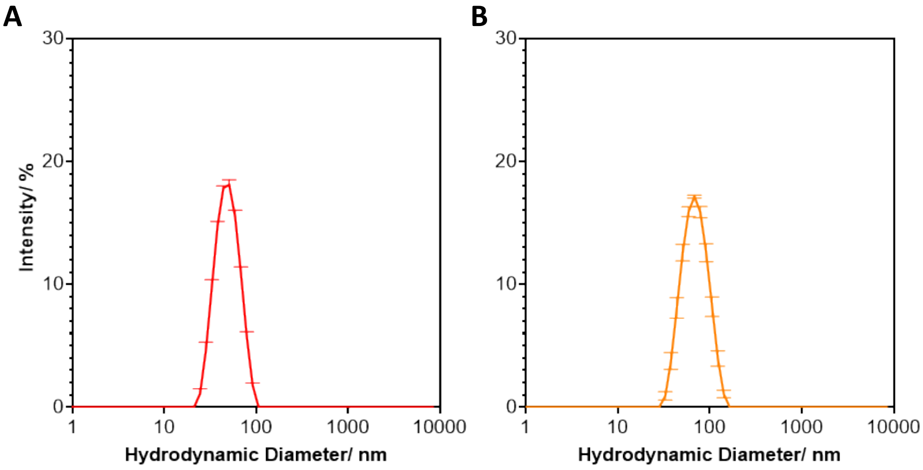

Figure S6: DLS of 40 nm AuNPs. **A:** DLS of bare 40 nm AuNPs, n = 3. **B:** DLS of 40 nm AuNPs detection probes, n = 3. Data shown as mean ± S.D.

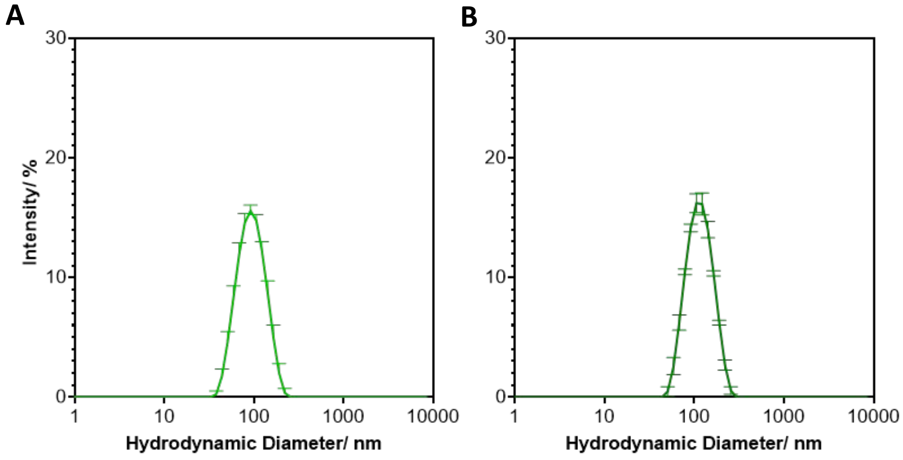

Figure S7: DLS of 80 nm AuNPs. **A:** DLS of bare 80 nm AuNPs, n = 3. **B:** DLS of 80 nm AuNPs detection probes, n = 3. Data shown as mean ± S.D.

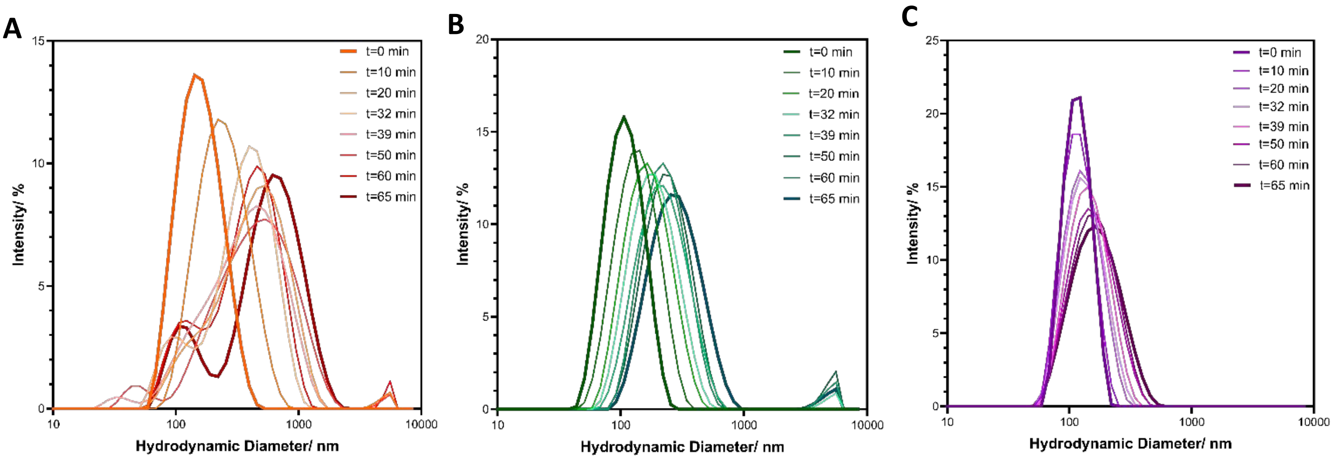

Figure S8: Intensity distribution of 40 nm AuNP, 80 nm AuNPs and PtNCs with SAPs (1:1 particle number ratio) over time. **A:** DLS of 40 nm AuNPs detection probes and SAPs (1:1 particle number ratio). **B:** DLS of 80 nm AuNPs detection probes and SAPs. **C:** DLS of PtNC detection probes and SAPs (1:1 particle number ratio).

Table S1: Extracted figures of merit from 4-parameter logistic regression fitting of plate-based assay using 40 nm or 80 nm AuNP detection probes. LC is the limit of the blanks. LD is the limit of detection in the signal domain. LOD lower and upper represent the 95 % confidence interval for the LOD.

Detection Probe	Amplification Probe	LC	LD	LOD/ ng mL^{-1}	LOD Lower/ ng mL^{-1}	LOD Upper/ ng mL^{-1}	RMSE in logY domain
40 nm AuNP	SAP	0.00038	0.00450	1.53	0.80	2.93	0.00325
80 nm AuNP	SAP	0.00378	0.00947	0.38	0.24	0.62	0.00349

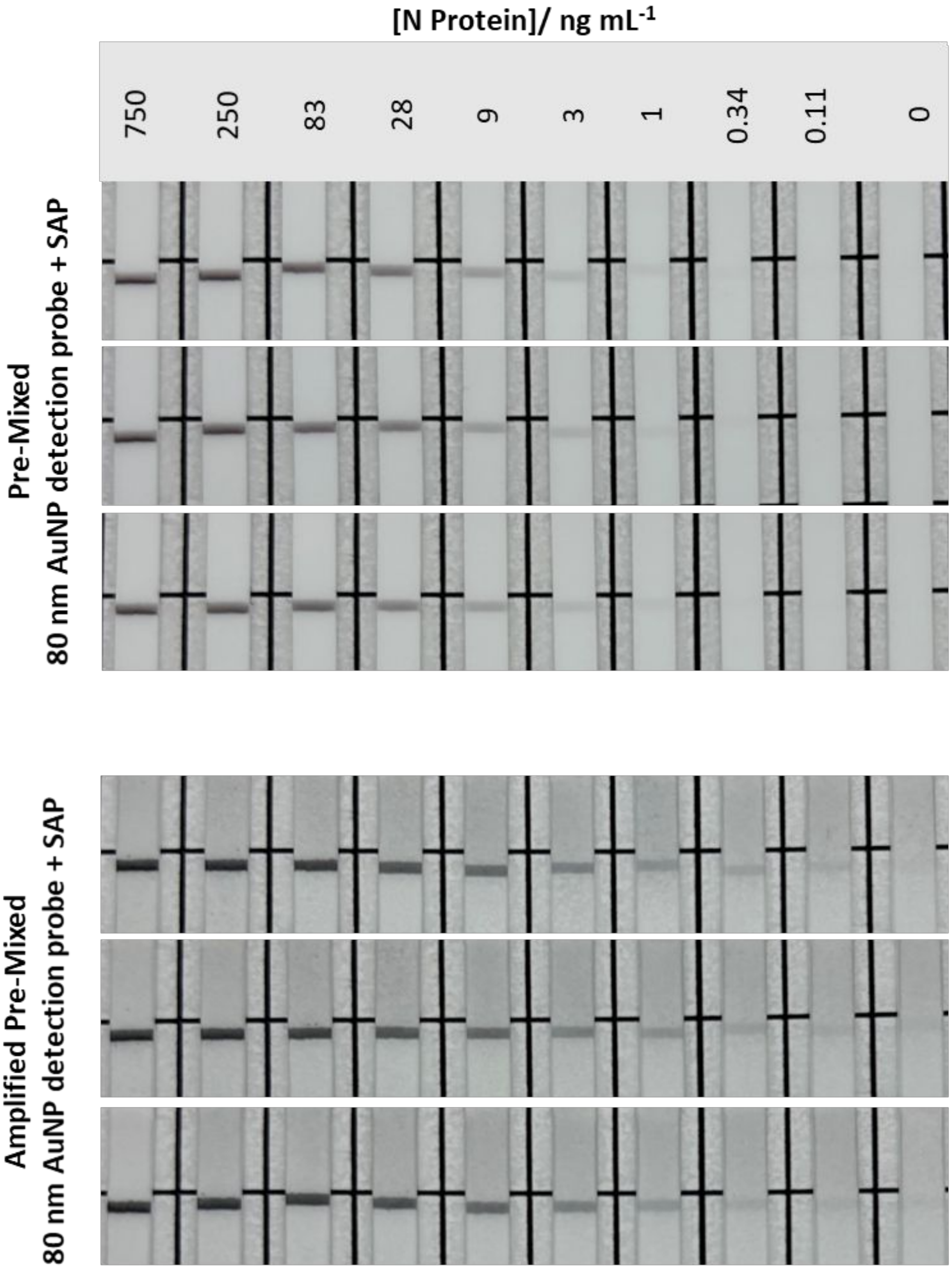

Figure S9: LFIA using 80 nm AuNP detection probes with a serial dilution of N protein antigen in running buffer. Top panel: Pre-mixed 80 nm AuNP detection probe and SAPs (1:6 particle number ratio), n = 3. Bottom panel: Pre-mixed 80 nm AuNP detection probe and SAPs after catalytical signal amplification, n = 3.

Table S2: Extracted figures of merit from 4-parameter logistic regression fitting of LFIA using pre-mixed 80 nm AuNP detection probes and SAPs (1:6 particle number ratio) with a serial dilution of N protein antigen in running buffer. LOD lower and upper represent the 95 % confidence interval for the LOD.

Assay Format	LOD/ ng mL^{-1}	LOD Lower/ ng mL^{-1}	LOD Upper/ ng mL^{-1}	RMSE in logY domain
80 nm AuNP only	1.68	0.86	3.26	0.00644
80 nm AuNP + SAP	1.21	0.61	2.41	0.00742
Amplified 80 nm AuNP + SAP	0.38	0.24	0.59	0.00791

Table S3: T-test comparing the extracted LODs from the 80 nm AuNP detection probe LFIA. Confidence level for the LOD interval set at 5%.

Data Set 1	Data Set 2	P-Value
80 nm AuNP only	80 nm AuNP + SAP	0.0509
80 nm AuNP only	Amplified 80 nm AuNP + SAP	0.000688

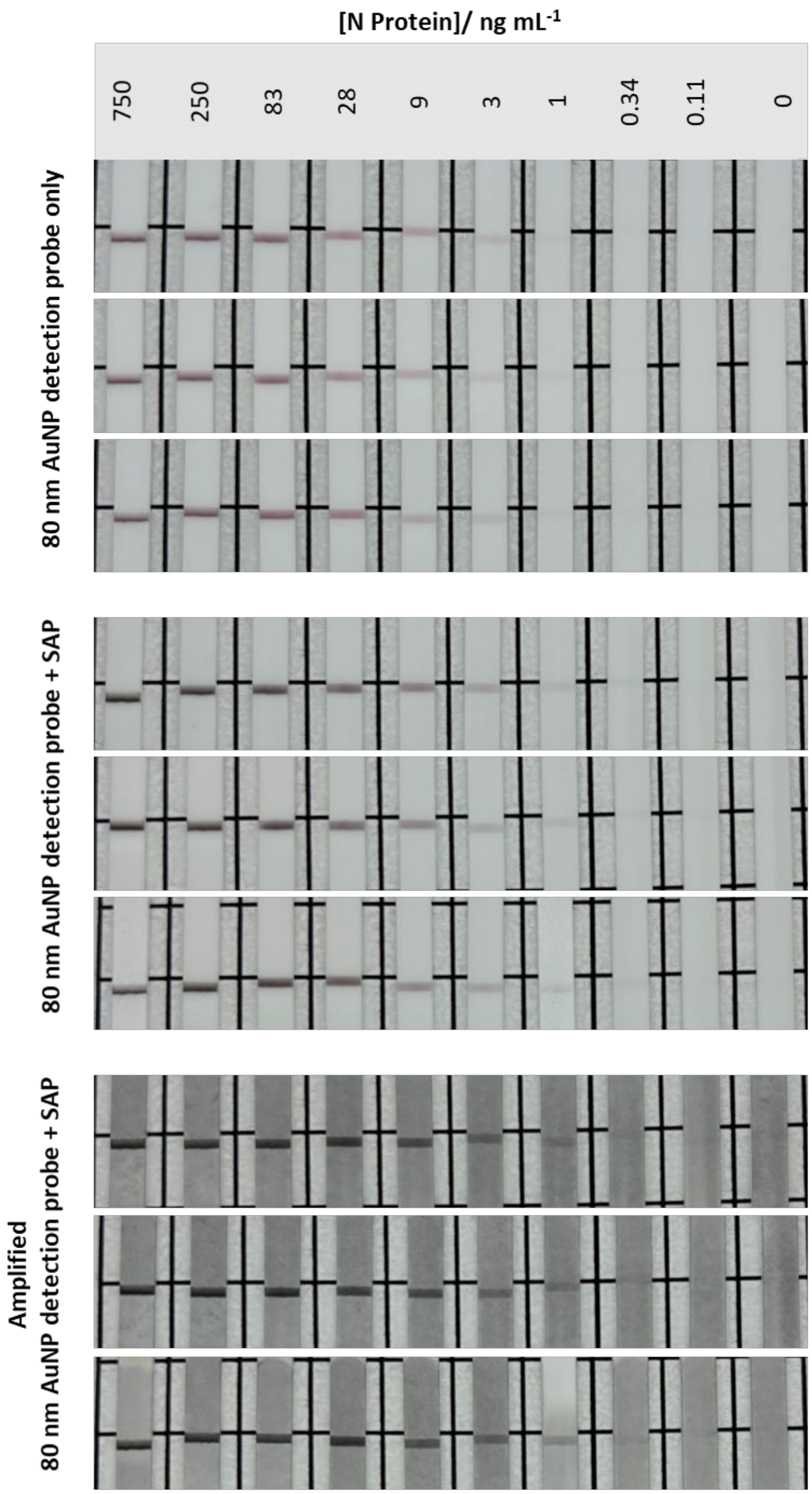

Figure S10: LFIA using 80 nm AuNP detection probes with a serial dilution of N protein antigen in running buffer. Top panel: 80 nm AuNP detection probe only, n = 3. Middle panel: 80 nm AuNP detection probe with sequential flow of SAPs (1:6 particle number ratio), n = 3. Bottom panel: 80 nm AuNP detection probe with sequential flow of SAPs after catalytical signal amplification, n = 3

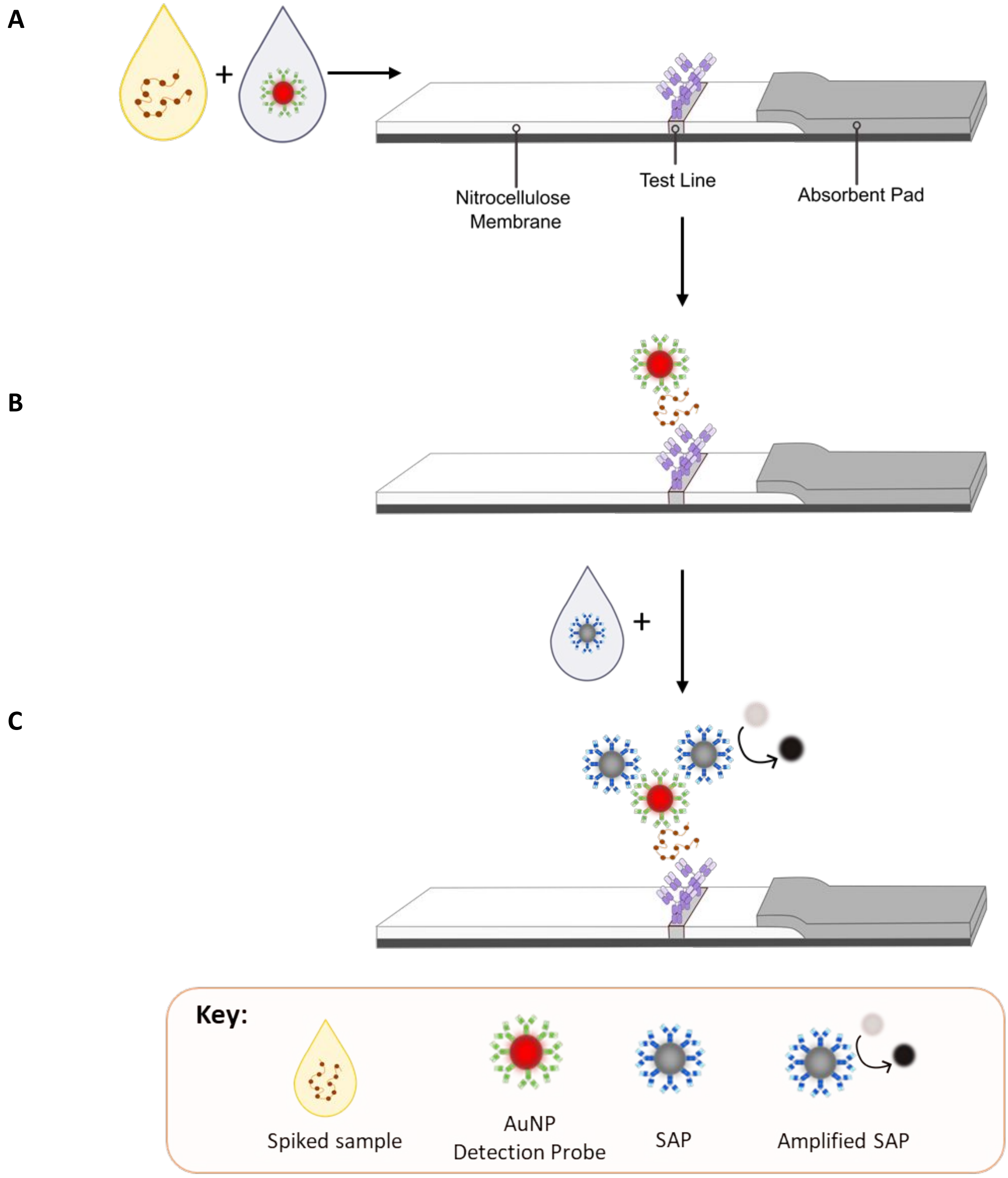

Figure S11: Schematic of half dipstick LFIA under the sequential nanoparticle flow regime. **A:** Addition of spiked sample and nanoparticle detection probe to the half dipstick LFIA strip. **B:** Formation of a sandwich complex and visible signal at the test line. **C:** Addition of the SAP and formation of nanoparticle network at the test line. The signal is enhanced through immobilization of a more nanoparticle probes at the test line and the inclusion of catalytically active nanoparticles.

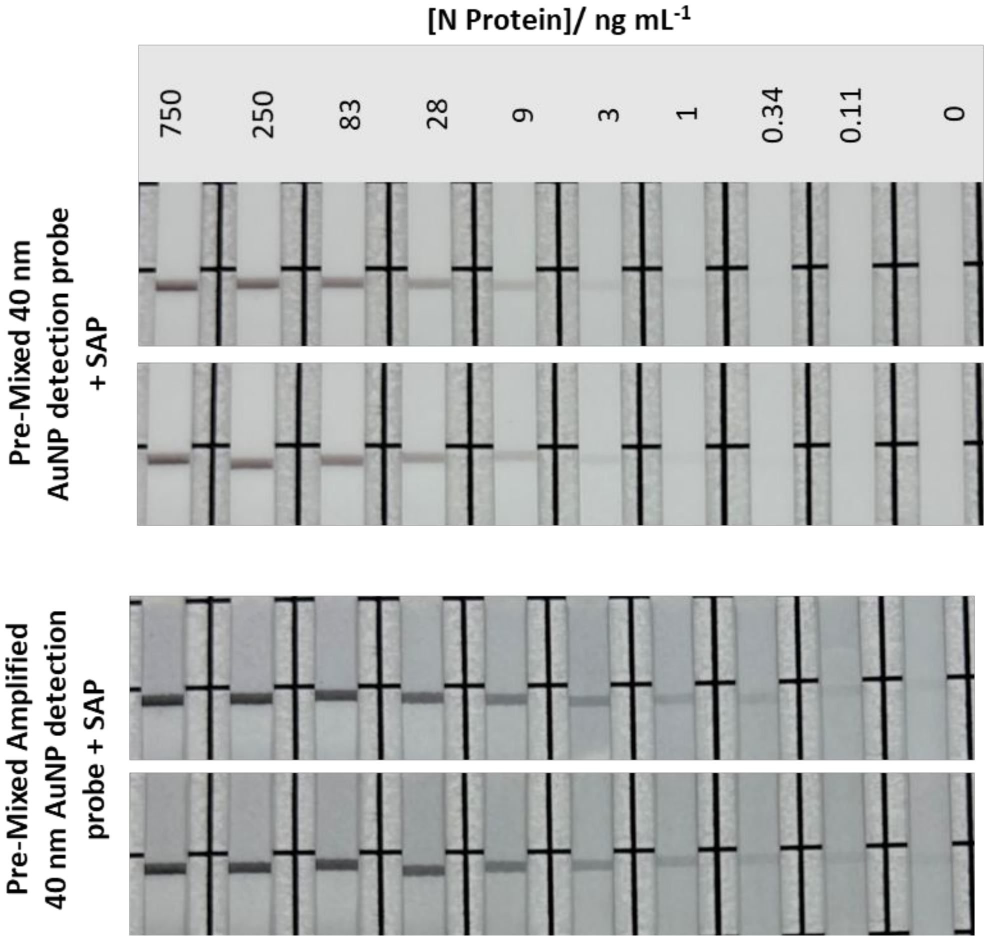

Figure S12: LFIA using 40 nm AuNP detection probes with a serial dilution of N protein antigen in running buffer. Top panel: Pre-mixed 40 nm AuNP detection probe and SAPs (1:3 particle number ratio), n = 2. Bottom panel: Pre-mixed 40 nm AuNP detection probe and SAPs after catalytical signal amplification, n = 2.

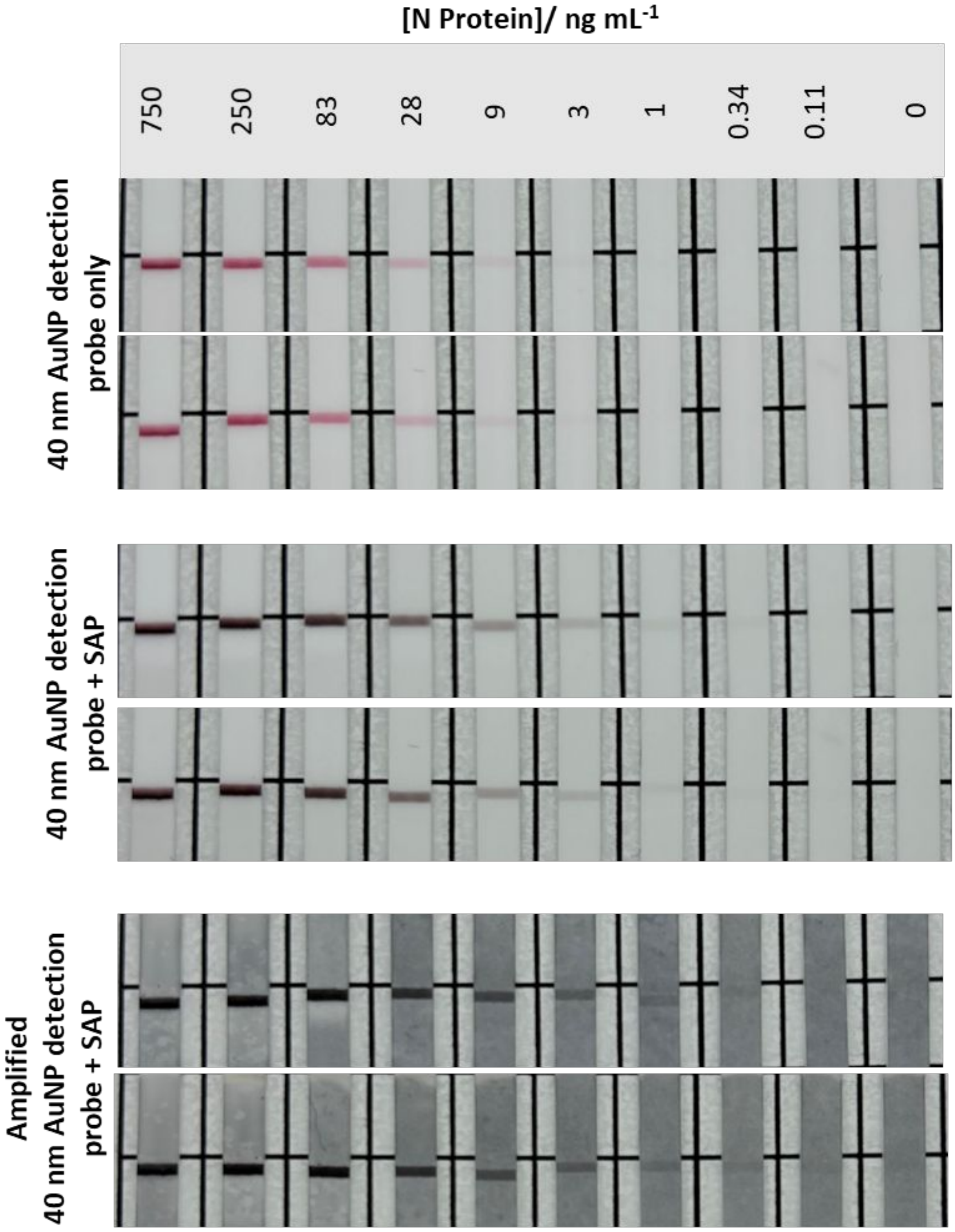

Figure S13: LFIA using 40 nm AuNP detection probes with a serial dilution of N protein antigen in running buffer. Top panel: 40 nm AuNP detection probe only, n = 2. Middle panel: 40 nm AuNP detection probe with sequential flow of SAPs, n = 2. Bottom panel: 40 nm AuNP detection probe with sequential flow of SAPs after catalytical signal amplification, n = 2.

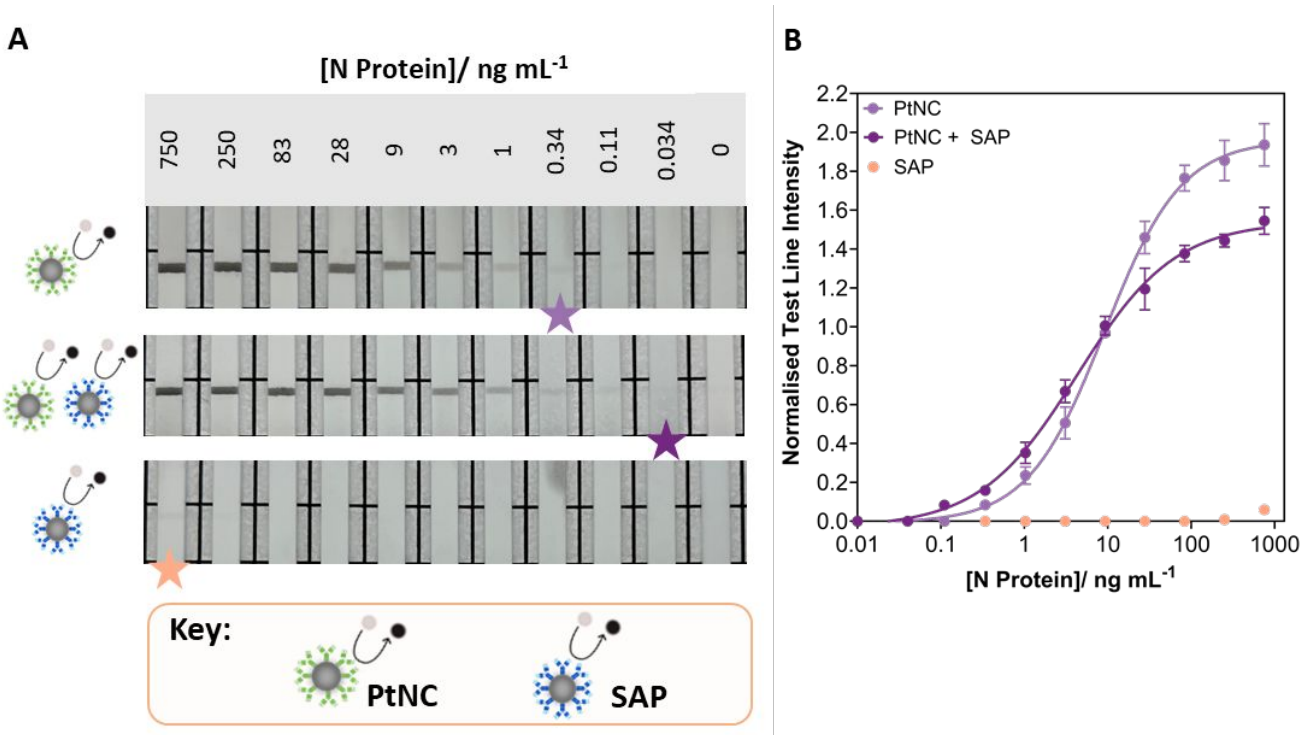

Figure S14: LFIAs utilizing network formation to induce signal enhancement. **A:** Representative images of serial dilution of N protein LFIA strips utilizing PtNC detection probes and SAPs (1:1 particle number ratio). Catalytic amplification was utilized to further enhance the signal. The assay was conducted in a spiked buffer sample matrix. The star on each set of LFIA strips represents the visual LOD. **B:** Extracted test line pixel intensity for N protein serial dilution with PtNC detection probes and SAPs, n = 3. Data shown as mean ± S.D.

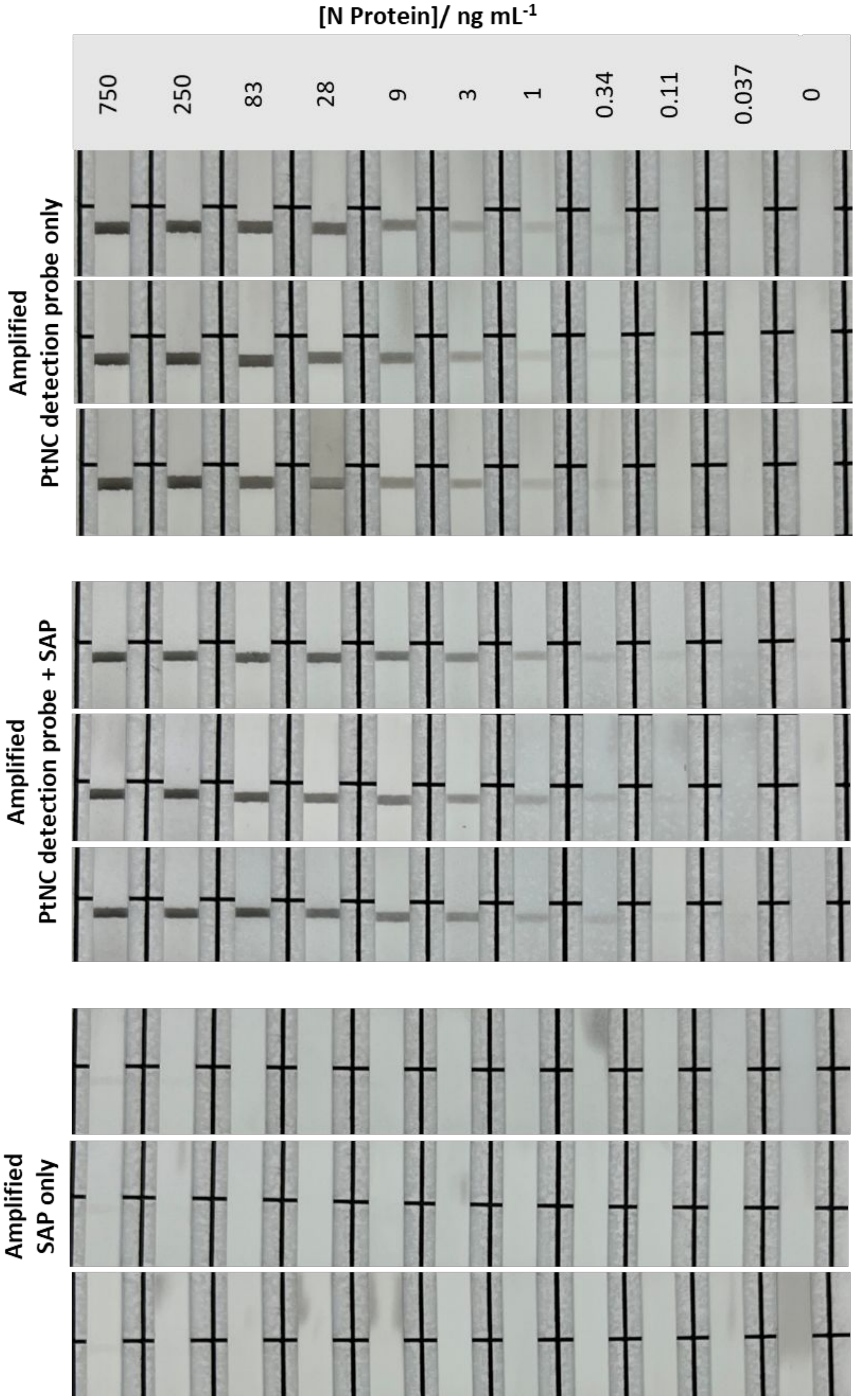

Figure S15: LFIA using PtNC detection probes with a serial dilution of N protein antigen in running buffer. Top panel: catalytically amplified PtNC detection probe only, n = 3. Middle panel: pre-mixed catalytically amplified PtNC detection probe and SAPs (1:1 particle number ratio), n = 3. Bottom panel: catalytically amplified SAPs only, n = 3.

Table S4: Extracted figures of merit from 4-parameter logistic regression fitting of LFIA using PtNC detection probes and serial dilution of N protein antigen in running buffer. LOD lower and upper represent the 95 % confidence interval for the LOD.

Assay Format	LOD/ ng mL^{-1}	LOD Lower/ ng mL^{-1}	LOD Upper/ ng mL^{-1}	RMSE in logY domain
Amplified PtNC only	0.38	0.14	1.04	0.01767
Amplified PtNC + SAP	0.14	0.075	0.26	0.00732

Table S5: T-test comparing the extracted LODs from the PtNC detection probe LFIA. Confidence level for the LOD interval set at 5%.

Data Set 1	Data Set 2	P-Value
Amplified PtNC only	Amplified PtNC + SAP	0.107

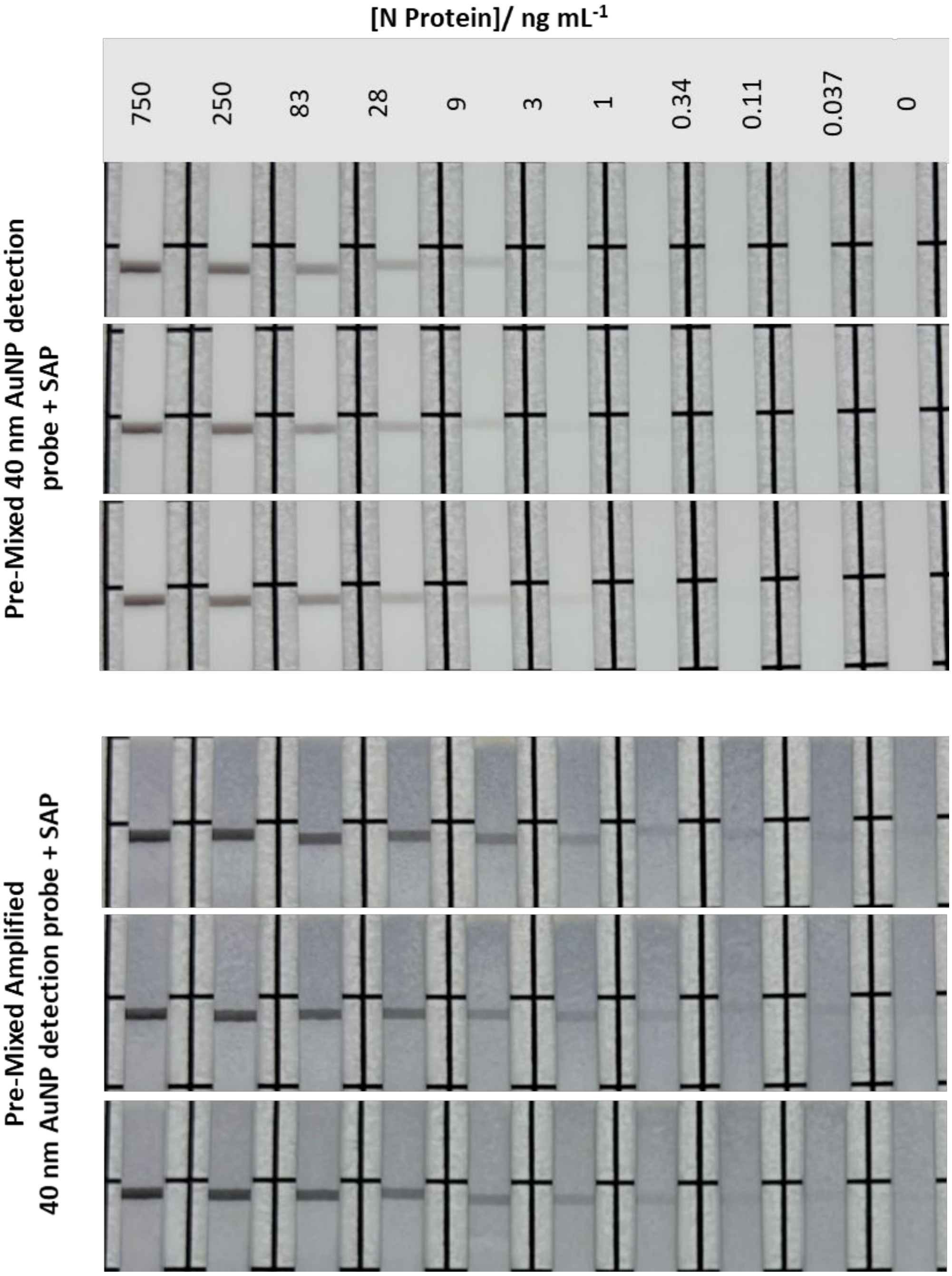

Figure S16: LFIA using 40 nm AuNP detection probes with a serial dilution of N protein antigen in spiked human saliva. Top panel: Pre-mixed 40 nm AuNP detection probes and SAPs (1:3 particle number ratio), n = 3. Bottom panel: Pre-mixed 40 nm AuNP detection probe and SAPs after catalytical signal amplification, n = 3.

Table S6: Extracted figures of merit from 4-parameter logistic regression fitting of LFIA using pre-mixed 40 nm AuNP detection probes and SAPs (1:3 particle number ratio) with a serial dilution of N protein antigen in saliva. LOD false negative and blank false positive rate set at 2.5 %. LOD lower and upper represent the 95 % confidence interval for the LOD.

Assay Format	LOD/ ng mL^{-1}	LOD Lower/ ng mL^{-1}	LOD Upper/ ng mL^{-1}	RMSE in logY domain
40 nm AuNP only	9.38	3.76	23.4	0.00360
40 nm AuNP + SAP	3.30	2.01	5.41	0.00507
Amplified 40 nm AuNP + SAP	0.22	0.033	1.38	0.00922

Table S7: T-test comparing the extracted LODs from the 40 nm AuNP detection probe LFIA. Confidence level for the LOD interval set at 5 %.

Data Set 1	Data Set 2	P-Value
40 nm AuNP only	40 nm AuNP + SAP	0.0558
40 nm AuNP only	Amplified 40 nm AuNP + SAP	0.00094

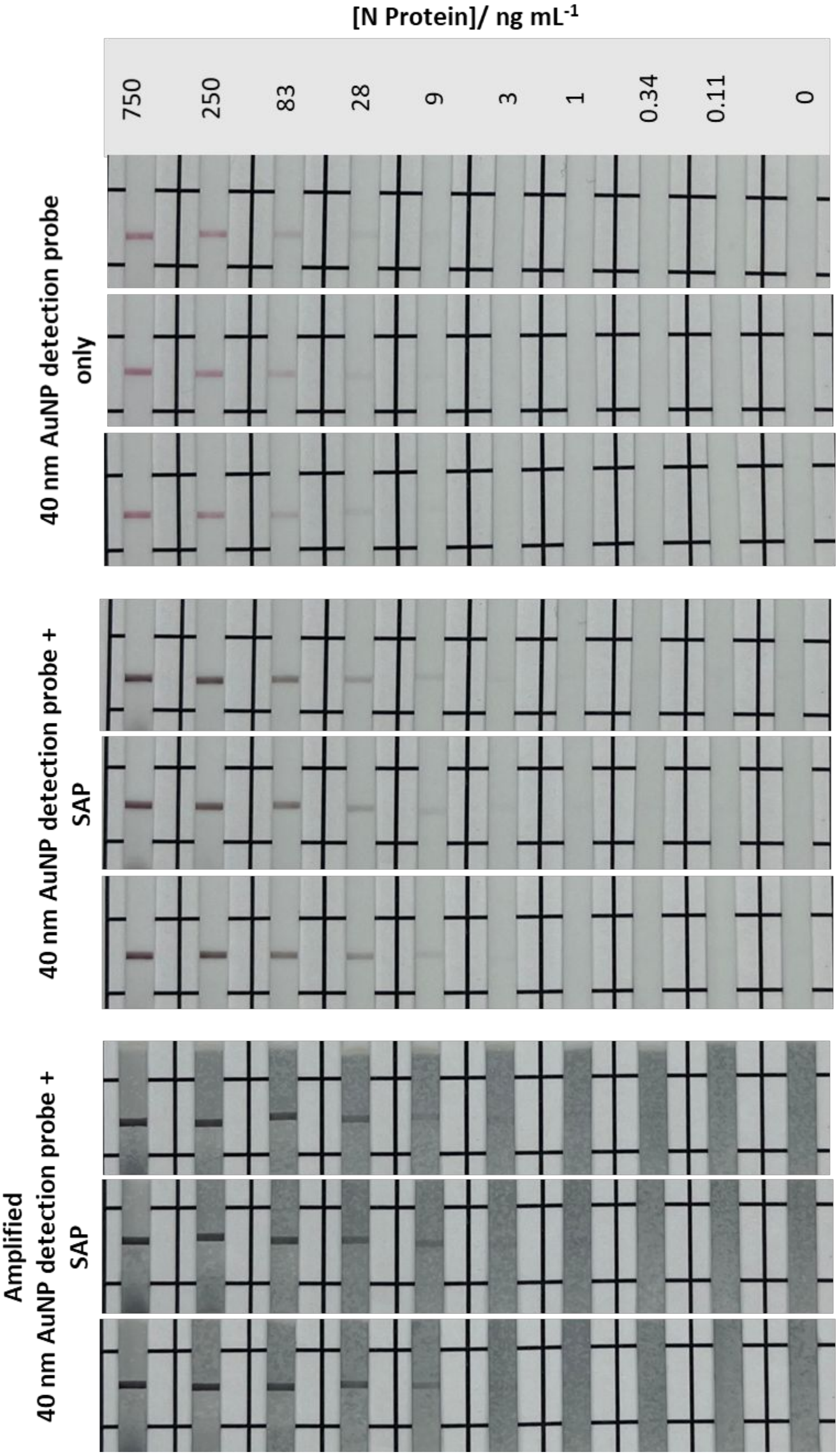

Figure S17: LFIA using 40 nm AuNP detection probes with a serial dilution of N protein antigen in spiked human saliva. Top panel: 40 nm AuNP detection probes only, n = 3. Middle panel: 40 nm AuNP detection probe and SAPs under sequential flow, n = 3. Bottom panel: 40 nm AuNP detection probe with sequential flow of SAPs after catalytical signal amplification, n = 3.

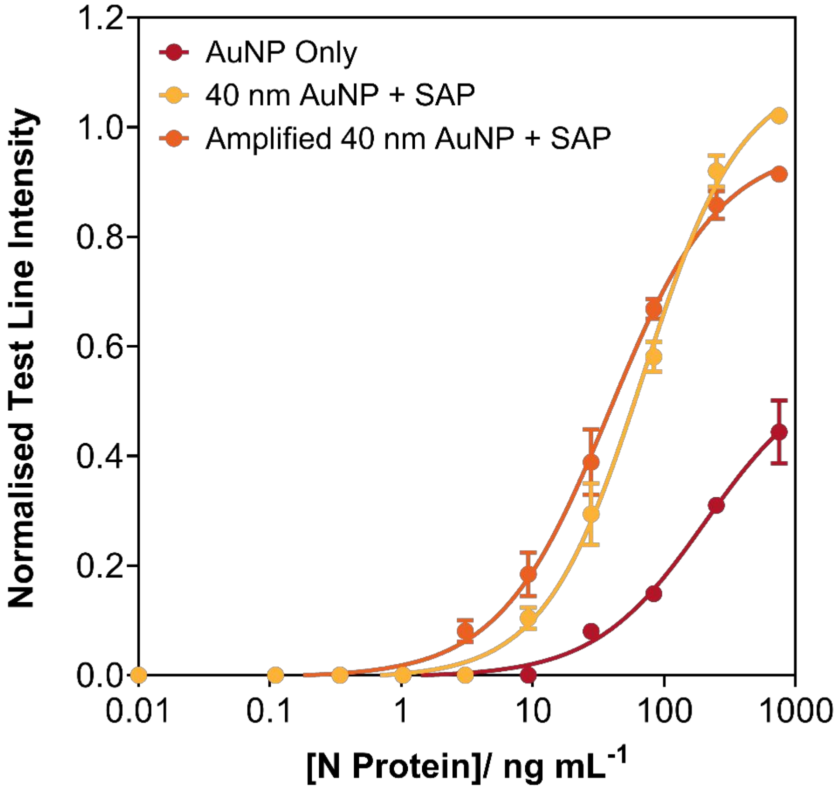

Figure S18: LFIA using 40 nm AuNP detection probes with a serial dilution of N protein antigen in spiked human saliva. SAPs are added under the sequential flow regime, before catalytic amplification. Extracted test line intensities are plotted as a function of N protein concentration using a 4-parameter logistic regression. Data shown as mean ± S.D, n = 3.

Experimental Section:

Assessment of Catalytic Activity of PtNCs and SAPs:

To the appropriate wells of a 96-well non-binding clear flat bottom polystyrene microplate (Corning), 10 µL of nanoparticle probe (PtNC or SAP; 0, 1, 2 or 4 pM) was added. 200 µL of 1-Step™ Ultra TMB ELISA Substrate Solutions was then added. The plate was added to a microplate reader (SpectraMax M5 microplate reader, Molecular Devices) and shaken for 5 s before measuring the OD at 652 nm for 30 min, reading at 30 s intervals.

Determination of Statistical Limit of Detection for Plate-Based Assays:

The limit of detection analysis for plate-based assays was extracted following previously reported methods (https://github.com/bensmiller/detection-limit-fitting).[1–4] Absorbance values (OD at 450 nm) were measured using the SpectraMax M5 microplate reader (Molecular Devices). Values were exported and experimental parameters assigned (assay format, N protein concentration). The N protein concentration and absorbance values were imported into the Detection Limit Fitting Tool and a 4-parameter logistic regression fit using the following equation:

$$y = \frac{a - d}{1 + \left(\frac{x}{c}\right)^b} + d$$

where y is the absorption signal, x is the N protein concentration, a is the asymptotes at the blank (background) signal, d is the saturation peak size, b the exponent defining the gradation of the region between the asymptotes (a and d), and c is the dissociation constant equivalent.[1]

The confidence levels (LOD false negative rate, blank false positive rate, variance outlier confidence level, and confidence level for LOD interval) were all set to 5 %.

Determination of Statistical Limit of Detection for Spiked Buffer LFIA Assays:

The limit of detection analysis for spiked buffer paper-based assays was extracted following previously reported methods (https://github.com/bensmiller/detection-limit-fitting).[1–4] Briefly, test line intensities were extracted using ImageJ as previously described. Test line intensities were normalized and were assigned experimental parameters (assay format, N protein concentration). The concentration and test line intensity values were imported into the Detection Limit Fitting Tool and a 4-parameter logistic regression fit using the following equation, as defined previously.

The confidence levels (LOD false negative rate, blank false positive rate, variance outlier confidence level, and confidence level for LOD interval) were all set to 5 %.

Determination of Statistical Limit of Detection for Spiked Saliva LFIA Assays:

The limit of detection analysis for spiked saliva paper-based assays was extracted following previously reported methods (https://github.com/bensmiller/detection-limit-fitting).[1–4] Briefly, test line intensities were extracted using ImageJ as previously described. Test line intensities were normalized and were assigned experimental parameters (assay format, N protein concentration). The concentration and test line intensity values were imported into the Detection Limit Fitting Tool and a 4-parameter logistic regression fit using the following equation, as defined previously.

The confidence levels for the LOD false negative and blank false positive rates set at 2.5 %. This differs from above to increase the confidence of the determined LOD in spiked saliva. The variance outlier confidence level and confidence level for LOD interval were set to 5 %.

Comparison of Limit of Detection Values: T-test

To compare the statistical significance between two calculated limit of detection values, a T-test was used. The analysis was performed in the *Detection Limit Fitting Tool* (https://github.com/bensmiller/detection-limit-fitting) software.[2] In each case, the limit of detection for the detection probe only assay was used a comparison dataset, since this was the previous gold standard assay. The confidence level for the limit of detection interval was set at 5%.

References:

(1) Miller, B. S.; Thomas, M. R.; Banner, M.; Kim, J.; Chen, Y.; Wei, Q.; Tseng, D. K.; Göröcs, Z. S.; Ozcan, A.; Stevens, M. M.; McKendry, R. A. Sub-Picomolar Lateral Flow Antigen Detection with Two-Wavelength Imaging of Composite Nanoparticles. *Biosens Bioelectron* **2022**, *207*, 114133. https://doi.org/10.1016/j.bios.2022.114133.

(2) Ben Miller. *Detection Limit Fitting Tool (https://github.com/bensmiller/detection-limit-fitting), GitHub.* 2022. https://github.com/bensmiller/detection-limit-fitting (accessed 2024-05-28).

(3) Holstein, C. A.; Griffin, M.; Hong, J.; Sampson, P. D. Statistical Method for Determining and Comparing Limits of Detection of Bioassays. *Anal Chem* **2015**, *87* (19), 9795–9801. https://doi.org/10.1021/acs.analchem.5b02082.

(4) Sadler, C. J.; Creamer, A.; Giang, K. A.; Darmawan, K. K.; Shamsabadi, A.; Richards, D. A.; Nilvebrant, J.; Wojciechowski, J. P.; Charchar, P.; Burdis, R.; Smith, F.; Yarovsky, I.; Nygren, P.-Å.; Stevens, M. M. Adding a Twist to Lateral Flow Immunoassays: A Direct Replacement of Antibodies with Helical Affibodies, from Selection to Application. *J Am Chem Soc* **2025**, *147* (14), 11925–11940. https://doi.org/10.1021/jacs.4c17452.
